# Screening for chlamydia and/or gonorrhea in primary health care: protocol for systematic review

**DOI:** 10.1186/s13643-018-0904-5

**Published:** 2018-12-26

**Authors:** Jennifer Pillay, Ainsley Moore, Prinon Rahman, Gabriel Lewin, Donna Reynolds, John Riva, Guyléne Thériault, Brett Thombs, Brenda Wilson, Joan Robinson, Amanda Ramdyal, Geneviéve Cadieux, Robin Featherstone, Anne N. Burchell, Jo-Anne Dillon, Ameeta Singh, Tom Wong, Marion Doull, Greg Traversy, Susan Courage, Tara MacGregor, Cydney Johnson, Ben Vandermeer, Lisa Hartling

**Affiliations:** 1 Alberta Research Centre for Health Evidence, University of Alberta, 11405 87 Avenue, Edmonton, Alberta T6G 1C9 Canada; 2 Department of Family Medicine, McMaster University, Hamilton, Canada; 3 Global Health and Guidelines Division, Public Health Agency of Canada, Edmonton, Canada; 4 Department of Family Medicine, University of Ottawa, Ottawa, Canada; 5 Department of Family and Community Medicine, University of Toronto, Toronto, Canada; 6 Department of Family Medicine, McGill University, Montreal, Canada; 7 Faculty of Medicine, McGill University, Montreal, Canada; 8 Community Health and Humanities, Faculty of Medicine, Memorial University of Newfoundland, St. John’s, Canada; 9 Division of Infectious Diseases, Department of Pediatrics, University of Alberta, Edmonton, Canada; 10 Dalla Lana School of Public Health, University of Toronto, Toronto, Canada; 11 Department of Microbiology and Immunology, University of Saskatchewan, Saskatoon, Canada; 12 Division of Infectious Diseases, Faculty of Medicine and Dentistry, University of Alberta, Edmonton, Canada; 13 Public Health Agency of Canada, Edmonton, Canada; 14 Ottawa Public Health, Ottawa, Canada

**Keywords:** Systematic review, Chlamydia, Gonorrhea, Screening, Sexually transmitted infections, Guideline

## Abstract

**Background:**

*Chlamydia trachomatis* and *Neisseria* g*onorrhoeae* are the most commonly reported sexually transmitted infections in Canada. Existing national guidance on screening for these infections was not based on a systematic review, and recommendations as well as implementation considerations (e.g., population groups, testing and case management) should be explicit and reflect the quality of evidence. The aim of this systematic review is to synthesize research on screening for these infections in sexually active individuals within primary care. We will also review evidence on how people weigh the relative importance of the potential outcomes from screening, rated as most important by the Canadian Task Force on Preventive Health Care (CTFPHC) with input from patients and stakeholders.

**Methods:**

We have developed a peer-reviewed strategy to comprehensively search MEDLINE, Embase, Cochrane Library, CINAHL, and PsycINFO for English and French literature published 1996 onwards. We will also search trial registries and conference proceedings, and mine references lists. Screening, study selection, risk of bias assessments, and quality of findings across studies (for each outcome) will be independently undertaken by two reviewers with consensus for final decisions. Data extraction will be conducted by one reviewer and checked by another for accuracy and completeness. The CTFPHC and content experts will provide input for decisions on study design (i.e., when and whether to include uncontrolled studies for screening effectiveness) and for interpretation of the findings.

**Discussion:**

The results section of the review will include a description of all studies, results of all analyses, including planned subgroup and sensitivity analyses, and evidence profiles and summary of findings tables incorporating assessment based on Grading of Recommendations Assessment, Development and Evaluation (GRADE) methods to communicate our confidence in the estimates of effect. We will compare our findings to others and discuss limitations of the review and available literature. The findings will be used by the CTFPHC—supplemented by consultations with patients and stakeholders and from other sources on issues of feasibility, acceptability, costs/resources, and equity―to inform recommendations on screening to support primary health care providers in delivering preventive care.

**Systematic review registration:**

International Prospective Register of Systematic Reviews (PROSPERO), registration number CRD42018100733.

**Electronic supplementary material:**

The online version of this article (10.1186/s13643-018-0904-5) contains supplementary material, which is available to authorized users.

## Background

### Background on infections

*Chlamydia trachomatis* (CT) and *Neisseria* g*onorrhoeae* (NG) are the most commonly reported bacterial STIs in Canada. Ten-year trends (2005-2014) in Canada indicate that the number of reported cases of CT infections has increased by 49% (206.0 to 307.4 per 100,000 [total population, not specific to sexually active individuals]), while reported cases of NG have increased by 61% (28.4 to 45.8 per 100,000) [1]. Although most individuals who are tested and found to be positive for genital CT or NG are reported, the true incidence of these infections is unknown for several reasons. Most infections are asymptomatic (with the exception of NG in males for which symptoms are more common) and, therefore, never tested and diagnosed unless complications arise. Treatment for many people follows syndromic diagnosis (i.e., treatment based on symptoms occurs without testing or waiting for test results), with variation between jurisdictions on whether or not these are reportable. Some higher risk individuals do not seek testing due to stigmatization. Further, these figures largely represent infections diagnosed at genital sites, even though studies have found relatively high rates of NG and CT infection at oropharyngeal and rectal (extragenital) sites. For example, reported rectal incidence rates in men who have sex with men (MSM) are 6-21% (NG) and 1-18% (CT), and in females attending sexually transmitted infection (STI) clinics and other high-risk settings are 0-3% (NG) and 7-17% (CT) [2–4]. In MSM, most extragenital infections occur in the absence of a genital infection (e.g., 91% for CT and 70% for NG [2]), whereas in women 9-29% of infections are single site anorectal infections without genital infection [2]. Extragenital infections are very often asymptomatic (e.g., anorectal < 5%) and found in the absence of reported risk behaviors, such as receptive anal and oral intercourse (i.e., influenced by reporting biases, contiguous spread of infection) [2, 5]. With increased testing at extragenital sites (e.g., in Quebec since 2014), when more recent (than 2014) data becomes available the rates of CT and NG will likely be higher yet.

Several risk factors and indicators are associated with differing prevalence of CT and NG infections (Additional file 1), including sex, age, geography, membership in a vulnerable group, high-risk sexual behaviors, and biological and epidemiological factors.

An estimated $51.4 million per year was spent on CT infections in Canada between 1991 and 2009, which included costs for screening, treatment, and long-term sequelae for untreated infection [6]. Costs specific for NG were not found, although a preliminary combined estimate for both direct and indirect costs of CT and NG (in 2000 CAN dollars) ranged from approximately $31.5 to $178.4 million [7]. The majority of costs related to CT and NG have been attributed to drugs (treatment of infections and complications), and acute-care hospital and physician costs, suggesting that much of the burden of these two infections can be reduced through implementation of effective prevention programs [7].

### Factors associated with rising incidence

The rise in CT and NG infections may largely be attributed to improved detection, rather than to an actual increase in incidence. This is attributable to higher diagnostic yield when using nucleic acid amplification tests (NAAT) instead of culture, higher testing volumes because of increased acceptability of NAAT testing (i.e., urine collection or, in women, self-collected vaginal swab versus clinician-collected urethral or cervical swab), and better targeting of screening to high-risk populations [8]. It may reflect to some extent more testing at extragenital sites. Increased incidence may also be attributed to some extent by more high-risk sexual behaviors [8]. There is also a hypothesis suggesting that the increased rates of CT may paradoxically be due to increased reinfection rates following aggressive control efforts (“seek and treat”), due to an “arrested immunity (from) the interruption of naturally acquired immunity associated with early initiation of treatment” [8]. This hypothesis was supported in British Columbia where intensive risk-based screening approaches, human immunodeficiency virus (HIV) infection and syphilis rates, and risk behaviors remained stable during 1996-2009 in the presence of increasing rates of CT. Although rates of CT and NG are increasing in Canada and many other countries, there have been stable or declining reported rates in their complications including pelvic inflammatory disease (PID) [1, 9–11]. A shift of PID management from hospital (where data on such complications are often collected) to out-patient settings [12, 13] may confound (underestimate) this reported complication rate to some extent. Additionally, the same aggressive control efforts for CT may also be arresting the underlying immune-mediated pathological processes that cause PID and ectopic pregnancy [8]. Nevertheless, preventing reinfection through successful treatment of sexual partners (“partner notification”) and treating reinfection early via retesting may be crucial to reducing infection rates, reinfection rates, and ultimately their complications. CT has a high frequency of transmission, with concordance rates of up to 75% of partners being reported [14].

### Consequences of CT and NG infections

In females, the infections with CT and NG can cause PID (infection/inflammation of the upper reproductive tract), chronic pelvic pain, ectopic pregnancy, and/or infertility. CT and NG are important causes of acute PID, with CT implicated in about one-fifth to one-third of all PID cases and about one-half in women aged 16-19 years [15–17]. Rates attributed to NG are not commonly reported, but PID may be attributed to NG more often than to CT; moreover, when from NG, PID may be associated with more severe symptoms and therefore discovered faster potentially leading to treatment and prevention of further complications such as ectopic pregnancy and infertility [18]. PID can be asymptomatic, especially when caused by CT. Rarely, other STIs (e.g., herpes simplex virus and trichomonas vaginalis) can cause PID [19]. Other causes of these complications include *Mycoplasma genitalium*, microorganisms associated with bacterial vaginosis, and respiratory and enteric pathogens that have colonized the lower genital tract [17, 18]. PID may resolve spontaneously, and it may be possible for the infections to cause ectopic pregnancy and infertility without first causing PID [9]. For example, the infections may be eradicated from the endocervix by the host immune response (“spontaneous resolution” in approximately half of cases at about 1 year after initial testing) [20], hence halting ascension of the infection, after the immune response has already triggered pathological processes in the fallopian tubes [9, 21].

Accurate rates of the above mentioned complications in cases of untreated infection are difficult to establish due to (i) diagnostic uncertainty for the infections (misclassification due to asymptomatic nature, previous reliance on culture for diagnosis which has poor sensitivity [missing cases]) and diagnostic uncertainty of the complications (PID diagnosis is usually clinical, rather than based on invasive and possibly inaccessible diagnostic laparoscopy, and neither sensitive nor specific), (ii) ethical and methodological issues with prospectively following untreated cases, as well as, (iii) the long duration of follow-up necessary to capture ectopic pregnancy and infertility consequences in relatively young populations having the highest prevalence of infection. Estimates of complication rates in females with untreated CT, relying on valid study designs (e.g., longitudinal cohorts and control arms of representative trials), are suggested to be in the range of 10-16% for PID [22, 23], 0.02-2% for ectopic pregnancy, and 0.1-4.6% for infertility [9]. Chronic pelvic pain may affect between a third and half of females with PID (thus 3-8% of those with infection) [9, 24]. The risks of PID and its sequelae may be higher when caused by NG (rates unreported) [18]. Apart from the incidence of these complications, the duration and severity of their effect varies (e.g., PID effects may be less or more severe, and may be of shorter duration than chronic pelvic pain) which may impact the importance people place on them [25].

In males, reproductive system complications include epididymitis, with or without orchitis, and, rarely [26], infertility. Extrapolating from a randomized trial of CT screening versus usual care in males aged 21-23 years in Denmark, the rate of epididymitis in untreated CT could be roughly estimated at 40 in 579 (7%), if CT was the major cause of epididymitis. This estimation was calculated from the number of people experiencing epididymitis at 12 months in the usual care group (40 in 9980; 0.4%) and the approximate number in this group having CT (i.e., 579), which (in absence of data) assumes a similar rate to that reported in the screening group (579 in 9980; 5.8%). The prevalence rate of CT in this trial agrees with those reported by population studies in Denmark [9], although most cases of epididymitis were identified using a proxy of doxycycline prescriptions in general practice, which may overestimate the CT-related incidence [27].

Other complications can occur in both reproductive (e.g. urethritis [males], cervicitis [females]) and non-reproductive sites (e.g., proctitis, pharyngitis, reactive arthritis, perihepatitis [Fitz-Hugh-Curtis syndrome in females]). Reactive arthritis (development of sterile inflammatory arthritis as a sequel to infection elsewhere, often in the gastrointestinal or urogenital tract) affects approximately 3-8% of people with a CT or NG infection, and in about 1-4% it will persist in the longer term (> 6 months) [28, 29]. An estimated 4-14% of patients with PID (possibly higher in adolescence) will experience Fitz-Hugh-Curtis syndrome. Although probably a necessary precursor to PID and its sequelae, approximately 85% of women with cervicitis have neither signs nor symptoms (4). An uncommon complication of NG in both sexes is disseminated gonococcal infection occurring in < 1% of patients, which is usually manifested by skin lesions, fever, arthralgia, acute arthritis and tenosynovitis, but may also lead to endocarditis, meningitis, sepsis and osteomyelitis [30]. Positive associations have been found between NG and prostate cancer (odds ratio [OR] with 95% confidence intervals [95% CIs]: 1.2 [1.1-1.4] [31] and 1.3 [1.1-1.5]) [31, 32] and between CT and cervical cancer (OR with 95% CI: 1.8 [1.0-3.0] independent of age and human papilloma virus status) [33] although incidence rates and causation are not easy to determine. Mortality has become a rare outcome, with estimates over the years per 100,000 in women ages 19-44 years decreasing from 0.3 deaths from PID alone in 1979 [34] to 0.1 deaths from CT and NG, PID, and ectopic pregnancy combined during 1999-2010 in the United States [15]. CT and NG may both increase the transmissibility of HIV, although findings are inconsistent, most studies have limitations (e.g., few have used actual HIV contact data), and large trials in countries with high HIV prevalence have failed to demonstrate that STI control interventions can reduce HIV incidence [1, 2, 35–38].

Recurring infections, or reinfection, increase the risk for complications [9, 39]. A meta-analysis of 38 studies found a reinfection rate for CT of 13.9% and for NG of 11.7% [39].

Little is known about the reproductive consequences from single-site extragenital CT infections, although it is understood that oropharyngeal infection can be transmitted to the genitals [40], and that infection of the genitals may occur through contiguous spread from extragenital sites [5].

### Consequences of screening, diagnosis and treatment

Screening, with the associated follow up including treatment, aims to reduce the consequences discussed above related to the natural course of infection. However, testing procedures themselves, inaccurate diagnostic tests, being diagnosed with an infection, and being treated with antibiotics may lead to other consequences that may be considered during decisions about screening.

#### Screening and diagnosis

Even though the diagnostic tests used for screening have good sensitivity and high specificity (see Additional file 2), some people will experience a false negative test—whereby treatment would not be provided and transmission to others may occur, or a false positive test informing them of an infection which does not exist. A false positive result may lead to adverse effects from treatment (see next section), and/or a risk for negative psychosocial effects about being infected with an STI (e.g., relationship stress), without any possible benefit to the individual tested. The availability of non-invasive diagnostic tests (urine, vaginal and rectal swabs), including self-sampling, reduces the likelihood of people experiencing discomfort or embarrassment during the procedure.

In those diagnosed with CT or NG, the benefits of treating the previously unknown infection and reducing risks for complications of the infection will be weighed by some individuals against the possible psychosocial effects of having an STI diagnosis. Also, STI stigma, caused by sociocultural norms (e.g., association with taboo and irresponsible or immoral behaviors) and intensified by institutional sources (e.g., media messages, fear-based education and prevention measures, judgemental attitudes of health care providers), can be a source of guilt, embarrassment, isolation, fear and distress [41]. Stigma hinders uptake of STI testing, disclosure and partner notification, treatment (seeking and adherence) and information seeking. A systematic review of qualitative studies on women’s experiences with CT screening found that most emotions about testing were negative, including fear, anxiety and embarrassment, although some were positive and related to a sense of self-care (“taking care”). A positive diagnosis often led to shock, blame, and anxiety for future reproductive health, relationship uncertainty, isolation and guilt.

Conversely, some felt relief at catching the infection or little concern because of thinking the infection is minor [42]. There appears to be negative and positive psychosocial consequences of both screening and receiving a positive diagnosis. Likewise, when considering quality of life and well-being, the possibility of a positive impact on these outcomes from reducing infection complications in some may be weighed against the possibility of negative impact from a positive diagnosis in others [25, 43]. Apart from psychosocial impacts, failure of screening programs (e.g., inadequate partner notification and treatment) to cure the infection or their possible adverse effect on immune processes (arrested immunity), as described above, may also increase chances for reinfection, which increase the likelihood of sequelae and additional transmission of the infection.

#### Treatment

Treatment for cure of CT and NG is effective (> 95% for CT and > 85% for NG, if uncomplicated infection) if properly adhered to, and will reduce the risk for complications of the infections as described above. Antibiotics typically used to treat CT and NG (described in Additional file 2) are quite commonly (15-25%) associated with mild adverse effects (AEs) including diarrhea, vomiting, constipation, abdominal pain, vertigo, fatigue and headache [44, 45]. The majority of AEs from CT and NG treatment are gastrointestinal in nature and may be severe in some cases particularly for NG where combination treatment or higher-dose single agents are used (e.g., 2 vs. 1 g dose of azithromycin). Very rarely (< 1 in 1000 people treated), people will have serious adverse drug reactions leading to hospitalization, from severe allergy to the antibiotic, *Clostridium difficile* colitis (possibly with life-threatening diarrhea), liver toxicity, heartbeat irregularities (from azithromycin although mainly for multi-day doses in specific patient subgroups), or other organ complications [44–48].

### Rationale for screening programs

Screening is a program, not only a test. Screening therefore includes a series of events initiated by offering of the test to diagnose an infection in those asymptomatic or not purposively seeking care for symptoms, detection of infection, with follow up for treatment and possibly partner notification and treatment, and retesting of cases to detect and treat reinfection [49].

While CT and NG may present with symptoms based on the location of infection, it is common that these STIs are detected asymptomatically. This increases both the risk of transmission to others and chances for complications when left undetected and untreated. The target groups for screening are usually defined by age and sex, considering prevalence and consequences of untreated infection. Further, although knowledge of behavioral and other risk factors (e.g., inconsistent condom use, multiple sex partners, MSM) will help identify those at a higher risk of becoming infected, there are challenges to accurate identification. People at high-risk may access services infrequently, they may not accurately self-report higher risk behaviors (e.g., because of stigma and often short recall period [e.g., couple of months]) which may lead to inaccurate reporting, results, and missing cases [2].

In the absence of treatment, infections persist for many weeks or months with the mean duration of CT from modeling estimated at 1.4 years [50] and NG commonly assumed to last approximately 6 months [51]. In women, treating the infections before their ascension from the lower to upper reproductive tract appears to be highly beneficial to prevent long-term sequelae [9]. Nevertheless, reductions in complications within screening trial participants for whom duration of infection is unknown and may be quite long suggests that screening and treating at variable durations of infection may be beneficial.

There are two possible goals of screening for NG and CT infections: *first*, to control the transmission and reduce the prevalence of the infection(s) in the population; and *second*, to reduce the risk of complications, especially reproductive tract complications in women [49]. The priority of these goals may influence what approaches are taken to screening. For example, coverage of a large proportion of the population may be necessary to reduce transmission and support population-based approaches. Without empirical data from randomized controlled trials (RCTs), a recent estimate based on several models found that screening all sexually active young adults (aged 16-44 years) at intervals of 2–5 years (corresponding to a yearly coverage of about 20% of this population) for 5–10 years could potentially reduce the prevalence of CT substantially (i.e., by at least 2-3 times) [52]. Screening to reduce serious complications may focus on opportunistic forms of screening where testing is offered to people in health care settings such as during visits to clinician offices or other health care sites including pharmacies [53] or emergency departments [54]. Other detection strategies focus on high-risk and/or hard-to-reach populations using outreach approaches in non-health community settings such as bars, sex venues, or mobile vans [55–57]. Testing may be provided to the entire population at risk (universal screening of all sexually active persons) or based on a strategy to target high-risk subpopulations.

The purpose of this review is to examine evidence on screening for *Chlamydia trachomatis* (CT) and *Neisseria* g*onorrhoeae* (NG) infections in sexually active individuals within primary health care. Specific rationale for developing this guideline, and recent national guidelines from other countries, are described in Additional files 3 and 4. The findings will be used by the Canadian Task Force on Preventive Health Care (CTFPHC)—supplemented by consultations with patients on outcome valuation and by information from organizational stakeholders and other sources on issues of feasibility, acceptability, costs/resources, and equity―to inform recommendations on screening to support primary health care providers in delivering preventive care.

## Methods/design

The Evidence Review and Synthesis Centre (ERSC) at the University of Alberta’s Alberta Research Centre for Health Evidence, will complete this review. The review will be developed, conducted, and prepared according to the CTFPHC methods [58] and this protocol follows reporting standards [59]. A working group of CTFPHC members (AM, GL, DR, GT, BT, BW, JR) and content experts (AB, JD, AS, TM) was formed for development of the topic, refinement of the key questions (KQs) and scope (i.e., population, interventions, comparators, outcomes, timing, setting [PICOTS]). CTFPHC members rated outcomes for their importance for creating a recommendation. The CTFPHC and content experts will not be involved in the conduct of the review including selection of studies and data analysis, but will comment on the draft report and provide input on the interpretations of findings. The Science Team of the Global Health and Guidelines Division at the Public Health Agency of Canada (PHAC) (PR, MD, GT, SC) provided assistance and input on CTFPHC methodological considerations during the topic refinement and development of the protocol; they also provided input on the protocol. Perspectives of patients and members of the public will be incorporated, regarding prioritization of outcomes for the final review. Any changes to the outcomes based on patient input will be reported in the final report. Stakeholder organizations (*n* = 14) reviewed the KQs and PICOTs and a draft version of this protocol was peer-reviewed. All comments were considered when finalizing this protocol. This final version of the protocol has been approved by the entire CTFPHC, and will be registered with the International Prospective Registry of Systematic Reviews (PROSPERO) database.

### Key questions

- KQ1: What is the effectiveness of screening compared with no screening for chlamydia and/or gonorrhea in non-pregnant sexually active individuals?
- KQ2: What is the comparative effectiveness of different screening approaches for chlamydia and/or gonorrhea in non-pregnant sexually active individuals?
- KQ3: What is the relative importance that people place on the potential outcomes from screening for chlamydia and/or gonorrhea?

### Analytical framework

**Fig. 1:**
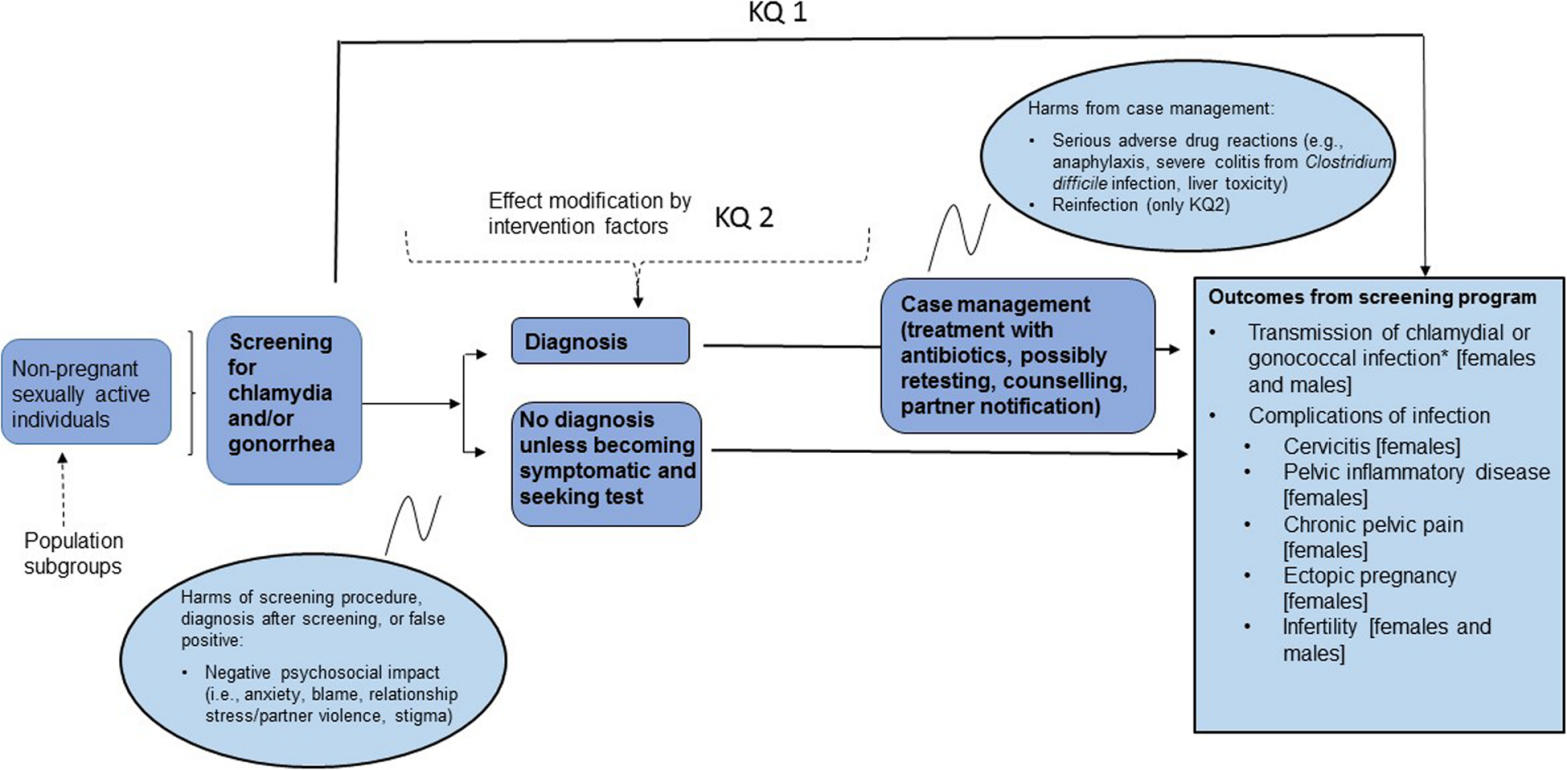
Analytical framework

Figure 1 depicts the relationship between the population, interventions, and outcomes of interest for this review.

### Eligibility criteria

Tables 1 and 2 outline each KQ’s study eligibility criteria (i.e., PICOTS).

**Table 1:** Eligibility criteria using PICOTS for Key Questions 1 and 2: Effectiveness and comparative effectiveness of screening approaches

Criteria	Inclusion	Exclusion
Population	**KQ 1 & 2**: Non-pregnant sexually active individuals Population subgroups: a. Population recruitment/identification strategy: clinician office (family doctor or pediatrician) vs. community health site (e.g., emergency room, school health clinic, pharmacy, sexual health/abortion/fertility clinics) vs. outreach program (e.g., field visits to homes, sex venues, bathhouses, homeless shelters, mobile vans, recreational or educational settings, online) vs. population register-based program not affiliated with health setting b. Demographics: age (10-14, 15-19, 20-24, 25-29, 30-49, 50+ years), sex (female vs male) c. Asymptomatic only (as determined by primary study authors) vs. all people not presenting with symptoms d. High risk individuals based on sexual behaviors and/or other factors, as defined by authors of primary studies	▪ Studies focusing on pregnant females ▪ Focus of study is on retesting cases, where all participants have recent diagnosis (≤12 months) of chlamydia or gonorrhea ▪ Studies focusing on those presenting with STI symptoms
Intervention	**KQ 1 & 2**: Any screening approach Intervention subgroup: Screening for chlamydia vs. gonorrhea vs. chlamydia and gonorrhea Screening may use any diagnostic test and treatment process for positive tests (e.g., referral to doctor, direct prescription), and may (but not necessarily) include partner notification/treatment and retesting of cases. If risk-based intervention strategy, may use any method to identify high-risk people. Sample may be collected by clinician or patient, and either on-site or at home. Postal delivery may be used for receiving or submitting screening tests.	• If focus is on re-testing/screening or testing partners • We will not exclude studies screening for CT and/or NG as well other STIs.
Comparator	**KQ1**: No screening **KQ2:** Any screening comparison differing from the intervention by the following factors: a. Universal vs. risk-based testing b. Health care setting only: sample collection location (i.e., clinic/health care setting vs. home) c. Outreach screening only: offered through street-based (e.g. mobile van) vs. other venues (e.g. bars, community services, bath houses, sporting events) d. Sample collection method (i.e., NAAT vs culture; invasive [urethral or cervical swab] vs non-invasive [urine or self-collected vaginal swab]; genital vs. genital and extragenital [e.g., as determined suitable]) e. Sample collection personnel (i.e., self vs. health care provider) f. Screening interval (i.e., one-time vs. annual vs. other) g. Case management approaches (i.e., retesting cases, method for partner notification/treatment) Studies from KQ1 may be used to help answer (indirectly) KQ2, for example when effectiveness appears to differ between different studies using different screening interventions compared with no screening.	
Outcomes	**KQ 1 and 2:** Primary Outcomes* a. Chlamydia/gonorrhea infection transmission: hierarchy using (i) incidence [# new cases during follow up/#population or person-years], (ii) prevalence [# positive tests/# in population at follow up time point], then (iii) index case management (as reported; could include # cases receiving treatment/# cases or also include partner notification and/or retesting/# cases) [females and males] b. Cervicitis [females] c. Pelvic inflammatory disease [PID; females] d. Ectopic pregnancy [females] e. Chronic pelvic pain (≥6 months duration) [females] f. Infertility: unable to conceive with unprotected sex for 12 months or longer [females and males] g. KQ2 only: Repeat infection/reinfection (proportion having positive test ≥3 months after the index infection; measurement may not distinguish between infection due to new exposure following treatment, treatment failure/nonadherence, false positives, or lack of initial treatment) h. Negative psychosocial impact (i.e., anxiety, sexual relationship distress including partner violence, stigmatization, blame) from screening procedure, or based on results a positive diagnostic test or presumptive diagnosis (i.e., regardless of test results in those with symptoms or considered at very high risk due to partner diagnosis) i. Serious** adverse drug reaction from antibiotic treatment (e.g., anaphylaxis, QTc interval prolongation/cardiac arrhythmias, severe colitis from Clostridium Difficile, hepatic toxicity, thrombocytopenia, hemolytic anemia; requiring hospitalization) **Secondary Outcomes:** Factors related to feasibility, acceptability, cost and process (from studies also reporting on one or more primary outcomes) • Feasibility (# tests returned/# invited) • Costs • Acceptability (testing process safe, valued, preference for type of provider, sampling, setting etc.) • Barriers to testing (any reason for not completing the testing procedure) • Treatment adherence: proportion cases not initiating or completing treatment as prescribed) • Partner treatment rate: proportion of index case sex partners treated • Retesting rate	
Timing	▪ Follow-up duration: Any duration, with exception of infection transmission and repeat infection (both ≥3 months), and as defined for outcomes of incidence of chronic pelvic pain (≥6 months) and infertility (≥12 months*)* ▪ Study publication: 1996 – present (post NAATs)	
Setting	▪ Any setting (indirectness to primary health care will be considered for studies where participant recruitment/identification strategy is undertaken in non-health care settings, but not criteria for inclusion) ▪ High and Very High Human Development Index countries http://hdr.undp.org/en/composite/HDI	
Study Design	▪ RCTs ▪ Non-randomized experimental studies (i.e., studies with intervention by investigators but without randomized allocation, e.g. quasi-randomized allocation) ▪ Controlled cohorts (prospective, retrospective, non-concurrent), controlled before-after studies, interrupted time series ▪ If feasible and if no or very low quality evidence from first 3 design categories for outcomes ‘h’ to ‘i’, we will look for evidence for these outcomes from uncontrolled cohorts or before-after studies with ≥30 participants or descriptive (e.g., qualitative, surveys) studies where participants have all had experience of screening. Reliance on controlled studies for outcomes ‘a’ to ‘g’ because of their relation to the natural history of the infections and therefore multiple potential confounders (e.g., multiple other causes of outcome) unaccounted for without a control group.	▪ Studies only published/available as conference proceedings or other gray literature (e.g., trial registry sites, government reports), unless information on study design (e.g., eligibility criteria, intervention and population description) is available (accessible online or via author contact) and sufficient for assessing quality/risk of bias.
Language	▪ English ▪ French	▪ Non-English/French articles

*An explanation of the process for rating outcomes for inclusion is in the text below Table 2

**Results in death or is life-threatening (i.e., requires inpatient hospitalization or results in prolongation of existing hospitalization; results in persistent or significant disability/incapacity; is a congenital anomaly/birth defect; is a medically important event or reaction) see https://www.ich.org/products/guidelines/efficacy/efficacy-single/article/clinical-safety-data-management-definitions-and-standards-for-expedited-reporting.html

**Table 2:** Eligibility criteria using PICOTS for Key Question 3: Outcome valuation

Criteria	Inclusion	Exclusion
Population	Non-pregnant sexually active individuals* Population subgroups: a) Population recruitment/identification strategy: clinician office (family doctor, pediatrician, nurse) vs. community health site (e.g., emergency room, school health clinic, pharmacy, sexual health/abortion/fertility clinics) vs. outreach program (e.g., field visits to homes, sex venues, bathhouses, homeless shelters, mobile vans, recreational or educational settings, online) vs. population register-based program not affiliated with health setting b) Current or previous infection with chlamydia or gonorrhea vs. not; current or previous experience of a primary outcome vs. not c) Demographics: age (10-14, 15-19, 20-24, 25-29, 30-49, 50+ years), sex (female vs male) d) Asymptomatic only (as determined by authors) vs. all people not presenting with symptoms e) High risk individuals based on sexual behaviors and/or other factors, as defined by authors of primary studies	▪ Pregnant women
Exposure	Experience with any screening program for chlamydia and/or gonorrhea; experience with infection or outcomes of interest; exposure to scenarios about screening process and possible outcomes of screening (benefits and harms) Focus of study is on consideration of possible, or assessment of definite, outcomes from screening. Studies of patients with outcomes (e.g., pelvic inflammatory disease) do not have to exclusively enroll patients with current or history of STIs.	
Comparison	Depending on study design, comparator may be no screening or another form of screening, or the study may not have a comparator. When only one arm (e.g. screening) of a comparative study is included in the assessment of patient preferences, this study will be classified as a non-comparative study.	
Outcomes	• Utilities/health state valuations • Non-utility, quantitative information on relative importance of benefits and harms (e.g., willingness to be screened, screening uptake, relative ratings/rankings, preference weights, willingness to pay, probability trade-offs) • Qualitative information indicating relative importance between benefits and harms All outcomes will only be in relation to the primary outcomes for KQ 1 and 2.	
Timing	▪ Follow-up duration: any or none ▪ Published: 1996 – present (post NAATs)	
Setting	▪ Any setting ▪ High and Very High Human Development Index countries http://hdr.undp.org/en/composite/HDI	
Study Design	▪ Any experimental or qualitative study design (e.g., stated and revealed preference studies [e.g. contingent analysis or valuation studies including discrete choice experiments, willingness to pay], studies directly [e.g., time-trade-off, standard gamble] or indirectly [mapping of health status instruments to quality of life scale] measuring health-state utility weights, surveys, qualitative studies)	▪ Studies only published/available as conference proceedings or other gray literature (e.g., government reports), unless information on study design (e.g., eligibility criteria, participant characteristics, presentation of scenarios) is available (accessible online or via author contact) and sufficient to assess methodological quality.
Language	▪ English ▪ French	▪ Non- English/French articles

*Studies that are reporting on health state values for people with experience of the outcomes of interest (e.g., PID) that may have been caused by another infectious source do not have to only include sexually active individuals

The population of interest for KQs 1 and 2 is non-pregnant sexually active individuals of any age. For KQ3, participants (i.e., patients, public) do not have to be sexually active if they have experienced one of the outcomes, such as PID, from another infectious source.

The most directly relevant screening approaches for this CTFPHC guideline are those delivered by primary health care providers, where participants are identified for screening via attendance at a clinic, or more systematic means (e.g., mailed invitation via health register), or some other form of screening offered by locations considered a first point of contact with the health system such as clinician offices (e.g., family physician, pediatrician, nurse practitioner) and community health settings (e.g., school health clinics, emergency departments, STI clinics, out-patient clinics, pharmacies, prisons, substance use clinics, family planning/fertility/abortion clinics, public health clinics). Screening undertaken in specialist settings (e.g., inpatient units, obstetrics/gynecology offices, infectious disease clinics), via outreach programming (e.g., sex venues, sports facilities, online), or using regional population register-based approaches (e.g., postal kits delivered to homes, not directly related to primary health care) is less directly relevant, but studies from these settings may inform the guideline and will be included.

For KQ2, comparing screening approaches, we may use direct and/or indirect comparisons. Direct comparisons are preferred, and come from studies having within-study, head-to-head comparisons of different screening approaches (e.g., home-based vs. clinic-based specimen collection in health clinic population, venue-based vs. clinic-based screening), while indirect comparisons can be made, cautiously, between studies where the interventions are different but there are similar comparators (e.g., comparing effects from two different screening programs [studies] each compared with no screening can be used to infer difference between the two screening programs).

Screening is a program, not only a test. Therefore, screening interventions only offering a test with communication of results to participants are not eligible. Interventions where the additional follow up is only a defined treatment referral, without active treatment provision and other activities such as retesting, partner notification, and/or post-test counseling, will be considered for inclusion if they report on one or more of our primary outcomes (e.g., number treated, psychosocial consequences, one or more of the included complications of interest).

#### Outcome rating

The preliminary outcomes of interest for this review are listed in Table 1. According to methods of Grading of Recommendations Assessment, Development and Evaluation (GRADE), the outcomes considered most patient-important and critical for making recommendations on screening for CT and/or NG were rated by members of the CTFPHC, and may be modified based on pending findings of an engagement exercise with a sample of sexually active individuals in Canada, conducted by an independent group with expertise in knowledge translation from St. Michael’s Hospital in Toronto, Ontario. All patient-important outcomes rated as critical (7 to 9 out of 9) and important (4 to 6 out of 9) are included, typically up to a *maximum number of seven*. This follows guidance based on cognitive limits when guideline panels are considering net balance of benefits and harms per question [60]. The CTFPHC working group rated several outcomes in males (e.g., epididymitis +/− orchitis) as being of lower importance than the outcomes listed in Table 1, and hence these are not included at this time. The outcomes related to *feasibility, acceptability, cost and process* will be considered secondary outcomes (not important or critical for decision making) and will primarily be used for implementation considerations during guideline development. Therefore, to be included in the review the studies must report on at least one or more of the primary outcomes, and findings for secondary outcomes will be drawn from these studies. All outcome ratings will be finalized prior to final study selection and data extraction; that is, the CTFPHC will be blinded to the studies and their results.

#### Additional eligibility considerations

We do not have a minimum threshold for study quality or inclusion criteria specific to items related to risk of bias (ROB), such as incomplete follow-up or lack of adequate allocation concealment. These factors will be taken into account when analyzing the data (e.g., possible sensitivity analysis) and interpreting the quality of evidence by outcome across studies.

For KQ1 and KQ2, we will not limit inclusion to only studies designed or analyzed using an intention-to-screen approach (e.g., including all patients *invited* to screen). Studies only using a per protocol design approach (e.g., only enrolling those actually tested) or analysis based on actual participation in screening will be included, but this distinction will be accounted for in the analysis and interpretation of the data (see Data Analysis and Synthesis). The decision to include uncontrolled studies for the outcomes of negative psychosocial impact and serious adverse effects of treatment will be based on the quality of the evidence from controlled/comparative studies. The decision will be made for each outcome-comparison of interest, including subgroups; for example, uncontrolled studies may only be included where controlled evidence is not found or is very low quality for certain populations (e.g., males) or intervention components (e.g., risk-assessment tool used for screening). We recognize that some outcomes (e.g., negative impact of diagnosis) may only be reported, regardless of study design, for screened participants even though they are also relevant to unscreened people. The CTFPHC and content experts will be involved in these decisions.

For assessing our comparison of universal versus risk-based screening approaches, we will include studies directly comparing universal versus risk-based screening strategies, but will also consider using indirect evidence between studies of universal screening and those using a risk-based approach only enrolling at-risk people (both versus no screening). The Additional file 5 describes and illustrates the ideal study designs for this comparison and some limitations when relying on other designs.

Case reports and case series (i.e., group of patients selected based on particular outcome) will be excluded, as will be papers not reporting primary research (e.g. editorials, commentaries, opinion pieces). Systematic reviews will not be eligible for inclusion, but will be examined and may serve to help identify additional relevant studies.

### Searching the literature

To build in efficiencies and capitalize on other work conducted, we are following the CTFPHC approach to integrating existing systematic reviews, where suitable (see Additional file 6). This approach focuses on examining existing high-quality reviews (key quality criteria being the ability of the search strategy and eligibility criteria to capture all relevant studies) in order to identify studies meeting our criteria, with the addition of an update of the evidence to the present date. The approach primarily uses the review to identify studies; we may also rely on review authors’ ROB assessments or extracted data (both pending quality checks and only if the tool covers the domains of interest [see Risk of Bias Assessment]), but will re-interpret all findings, including assessment of the quality of the body of evidence. This approach is particularly suitable for reviews when all, or a portion of (e.g., studies of a certain design) a KQ is covered by the studies in the available review. A comprehensive search for systematic reviews on this topic was conducted, with careful inspection of potentially suitable reviews for use with this approach. None were considered suitable for KQ 1 or 2 due to differing populations (e.g., reviews that excluded studies with participants that may have had symptoms), interventions (e.g., screening for CT with or without NG, but not only for NG), and settings (e.g., no inclusion of non-health care settings). Hence, a full de novo search is planned for KQ 1 and 2. Of note, our evidence review will differ in some aspects from the one used to inform the guideline of the United States Preventive Services Task Force (USPSTF) guideline [61]. Only studies or analyses where all participants were asymptomatic were included by the USPSTF, rather than including studies that also tested symptomatic individuals (who were not seeking care for symptoms). Moreover, the CTFPHC outcomes of interest differ to some extent, and it is unclear if studies in all settings defined by our definition of primary care were eligible in the USPSTF review. The CTFPHC is also interested in examining evidence about screening in specialist and non-health settings to help inform their recommendations.

For KQ3, we identified one systematic review [25] on valuing health states that will be used to answer the portion of this question that is related to people who have experienced the outcomes of interest (e.g., not a screened population and not necessarily due to CT or NG infection). This will enable us to focus our own full search on studies about screening for CT and/or NG, which will also capture other studies relevant to different portions of KQ3 (e.g., valuing complications of CT and NG among people screened or diagnosed with CT or NG but not experiencing the outcomes). Accordingly, we have conducted one search to capture studies for KQ1, KQ2, and a portion of KQ3, and another search to update the evidence from the integrated systematic review to help answer KQ3.

The literature search strategies have been developed and implemented by a research librarian. They consist of both controlled vocabulary, such as the National Library of Medicine’s MeSH (Medical Subject Headings), and keywords, and have been peer-reviewed using the Peer Review of Electronic Search Strategies (PRESS) checklist [62]. Because the integrated review on health state valuations also included studies of economic evaluations, we have modified the authors’ search slightly before updating this to the present date (2014 onwards). Searches are being restricted by language to include full texts published in English or French. Literature suggests language restrictions in systematic reviews on conventional medicine topics do not appear to bias results from meta-analyses [63, 64].

We have conducted (May 31-June 5, 2018) comprehensive searches in relevant bibliographic databases: Ovid Medline (1946-); Ovid Embase (1996-); Wiley Cochrane Library (inception-); CINAHL via EBSCOhost (1937-); and Ovid PsycINFO (1987-) (Additional file 7). Additional search sources will include trial registry records via ClinicalTrials.gov, meeting abstracts via the Conference Proceedings Citation Index – Science edition (Clarivate Analytics), and invitations to Canadian organizational stakeholders and content experts to submit reports/studies or identify websites for searching.

Reviewing the bibliographies of included papers and relevant systematic reviews will supplement the searches. We will contact authors (by email with three attempts over one month) of relevant protocols or trial registries not containing data, to obtain any reports or publications of completed studies. We will also contact authors of studies that are only reported in conference abstracts, reports, and other sources of information (e.g., trial registry sites) where full study details and where peer-review of the results have not been undertaken, to try to obtain enough information to include these studies (i.e., if we can adequately assess their study quality and characterize their PICOTS). Tables 1 and 2 contain our criteria for including studies reported in abstracts and other “gray literature”. Our data analysis section also describes how we will handle these studies.

The bibliographic database searches for all KQs will be updated approximately 4-5 months prior to publication date of the CTFPHC Guideline to identify any new studies.

All results of the database searches will be imported into an EndNote® database (Thomson Reuters, New York, NY) for reference citation and removal of duplicates by the librarian, and into DistillerSR (Evidence Partners Inc., Ottawa, Canada) for screening and selection procedures. Our supplementary search process will be documented (e.g., websites, search terms, dates) and any results passing an initial screen will be entered into Endnote and DistillerSR for full text review.

### Screening and selecting studies for inclusion

For the database searches, two reviewers will independently screen the titles and abstracts (when available) using broad inclusion/exclusion criteria. Citations will be classified as “include/unsure,” “exclude,” or “reference” (i.e., conference abstracts, protocols, and systematic reviews). One reviewer will review the “reference” group and will screen results of the supplementary searches (e.g., trial registry sites). The full text of all studies classified as “include/unsure”, identified through review of the reference citations, or screened as relevant from the supplementary searches will be retrieved for full review. Two reviewers will independently assess eligibility of full texts using a standard, piloted, form that outlines the inclusion and exclusion criteria. Disagreements on final inclusion of all studies will be resolved through consensus or a third reviewer. The title/abstract screening and full-text selection processes will be conducted and documented in DistillerSR. We will contact authors via e-mail (3 times over one month) when the details necessary to decide on inclusion have not been adequately documented in the publication. The flow of literature and reasons for full text exclusions will be recorded in a PRISMA Flow Chart, and for each study in an excluded studies list.

### Data extraction and reporting

We will use piloted, standardized data extraction forms. One reviewer will independently extract data from each included study into DistillerSR; a second reviewer will verify all data for accuracy and completeness. Disagreements will be resolved through discussion or a third reviewer.

For each key question, we will extract data on the following:

- author(s) and publication date
- funding source
- country of origin
- design and power calculation
- number of participants: assessed for eligibility, allocated to/receiving each intervention, screened [at each round, if applicable], retested, assessed for each outcome
- population(s): eligibility criteria, recruitment strategies, and participant baseline characteristics related to subgroups in Tables 1 and 2
- intervention(s)/exposure(s): screening for CT only, CT and NG, or NG only; risk stratification method (if used), diagnostic test, all reported case management activities, intervention factors listed in Table 1 (e.g., screening interval, personnel, other STIs), co-interventions; for KQ3: presentation or scenarios of outcomes from screening, if applicable
- comparator(s): KQ1, any details about usual care; KQ2, see intervention(s)
- setting(s): including locations of recruitment, screening, case management, other follow-up activities
- outcome measures: name, definition, measurement (i.e., tools, including scale and thresholds where applicable; diagnostic criteria) and ascertainment (e.g., health records and/or self-report), time point(s), as reported by studies
- details of analysis, including adjusted and sub-group analyses
- results (see elaboration below)

When there are multiple publications associated with a study we will consider the earliest report of the main (primary) outcome data to be the primary data source. We will extract data from the primary source first and then add outcome data reported in the secondary/associated publications and data sources. We will reference the primary source throughout the evidence report, but will also cite all associated literature that provided information. We will contact authors of included studies via email (with 3 contacts over one month) for clarification of study, participant, and result details.

We will record intention-to-screen results whenever possible, while recording the number in each arm with missing data. For dichotomous outcomes, we will record counts or proportions, and sample size, by study arm. Only numerical data for outcomes will be extracted; that is, we will make no assumptions on lack or presence of an outcome if this is not reported. If counts by group are not reported we will record the computed effect estimate provided by the author (e.g., RR, OR). If ORs are unadjusted and the sample sizes by group are reported, we will calculate the RR; we may also use the OR as an approximation of the RR if events rates are very low (< 5%). For continuous outcomes measures, we will extract (by arm) the mean baseline and endpoint or change scores, standard deviations (SD) or other measure of variability, and number analyzed. We will not include outcome data from studies that did not provide a follow-up change or endpoint score, or did not provide data/figures that could be used to calculate follow-up scores. If necessary, we will approximate means by medians. If SDs are not given, they will be computed from *p*-values, 95% confidence intervals (95% CIs), standard errors, z-statistics, or t-statistics. If computation of SDs is not possible they will be estimated from upper bound p-values, ranges, inter-quartile ranges, or (as a last resort) by imputation using the median SD from the other studies reporting on the outcome. When computing SDs for change from baseline values, we will assume a correlation of 0.5, unless other information is present in the study that allows us to compute it more precisely. Authors that report only p-values or narrative findings (e.g., “fewer”, “no difference”) will be contacted (3 times over 1 month) to obtain more specific data, although these studies will still be included when no additional data are obtained, and their results interpreted. We will use information from figures if no numerical values are provided; we will use available software (e.g., Plot Digitizer, http://plotdigitizer.sourceforge.net/) with agreement between two reviewers. We will, if feasible, accept individual patient data and conduct our own analysis.

Any relevant section of the results section of qualitative studies will be pasted into a Microsoft Excel spreadsheet for further analysis.

Data on within-study analysis for our subgroups of interest will be collected, including: subgroups (independent variables), the type of analysis (e.g., subgroup/stratified or regression analysis), the outcomes assessed (dependent variables), and the authors’ conclusions. We will collect data suitable for all patient and intervention subgroups (see Table 1) for performing our own subgroup analyses (e.g., stratified analysis, meta-regression) based on study-level data.

We will provide a narrative summary and tables describing the characteristics of all included studies. When possible, we will enter results from studies into Review Manager 5.3 and provide plots of the study results (regardless of decision to meta-analyze); otherwise results will be tabulated.

#### Unit of analysis issues

Unit of analysis errors can occur in studies that employ a cluster design (i.e., a clinical practice, school or community) and yet are analyzed at the individual level (i.e., patients), leading to overly precise results and contributing greater weight in a meta-analysis. Moreover, additional biases associated with clustering in this context occur for some outcomes. For example, when screening for STIs is undertaken in geographic clusters, the intervention in a cluster may not only affect the participants, but also their partners and others in their sexual network (indirect effects) which may reduce the level of re-exposure and overall rates of infection in a cluster [49]. For trials that recruit by cluster, we will perform adjustments for clustering if this was not done in the published report. We will calculate the “effective sample size”, which accounts for the design effect of the unit of analysis and will be based on the average cluster size and intraclass coefficient [65]. We will use an ICC of 0.028 [66].

### Risk of Bias assessment

Two reviewers will independently assess the ROB of each included study, with disagreements resolved through discussion or a third reviewer. The results for each study and across studies will be reported by each domain. The ROB for each study will be assessed on an outcome basis where needed, particularly when different outcomes are assumed to have different susceptibilities to bias; for example, self-reported outcomes are more prone to bias from non-blinding than objective outcomes. Outcomes at different time points may also differ in their ROB.

RCTs and controlled experimental studies (theoretically only differing from RCTs by lack of random sequence generation and not in other ROB domains) will be appraised using the 2011 version of the Cochrane Risk of Bias tool [65]. For non-randomized trials, we will add an additional assessment of selection bias (e.g., allocation method unrelated to characteristics associated with the outcomes) using a checklist developed by the National Institutes for Health and Care Excellence [67], such that some of these studies may receive an unclear rather than high ROB rating for sequence generation. Our assessments will consider the extent to which the possible biases may, or may not, have a meaningful impact on the direction or magnitude of the study findings [65].

Controlled observational studies will be appraised using the Newcastle-Ottawa Quality Assessment Scale [68]; three domains (sample selection [4 items], comparability of cohorts [1 item], and assessment of outcomes [3 items]) are evaluated. We will also report, separately, our assessment of the potential for selective outcome reporting for these studies; although protocols for observational studies are not often registered or published (limiting comparison of predetermined and reported outcomes and analysis), selective reporting may be at risk, such as when an outcome that is considered to have high importance for the topic and for patients is not addressed in the study.

Critical appraisal tools from the Critical Appraisal Skills Programme [69] and the Centre for Evidence-Based Management [70] will be used for qualitative and cross-sectional/survey studies, respectively. We will not use a specific tool for utility/preference-based studies but rather comment on key study characteristics, which may be associated with biased results (e.g. accounting for confounders, representativeness of population, inclusion of all outcomes in scenarios, presentation of outcomes in unbiased way [e.g., absolute effects]) [71].

Our assessments of the risk of bias will be incorporated into our assessment of the quality of the evidence across studies for each outcome (see Assessment of the Overall Quality of the Evidence using GRADE).

### Data analysis and synthesis

We will provide summaries of intervention effects for each study by calculating the appropriate statistics based on types of outcomes.

#### Key questions 1 and 2

For pairwise meta-analysis in KQs 1 and 2 (for all primary outcomes), because of anticipated between-study heterogeneity we will employ the DerSimonian Laird random effects model using Review Manager Version 5.3 (The Cochrane Collaboration, Copenhagen, Denmark). For dichotomous outcomes, we will report relative risks (RR) between groups with corresponding 95% CIs. For continuous outcomes, we will report a pooled mean difference (MD) when one measurement tool is used, or a standardized mean difference (SMD) when combining two or more outcome scales measuring similar constructs (based on clinical input). If we are not able to use a study’s data in a meta-analysis (e.g., only adjusted ORs or *p* values are reported), we will comment on these findings and compare them with results of the meta-analysis.

For outcomes having statistically significant effects, we will calculate absolute risk reduction (ARR) or number needed to screen (NNS) based on comparison with the median control group event rates and RR. We also anticipate reporting estimates of absolute effects for some of our age and our sex subgroups, at a minimum. Age categories that are unlikely to differ greatly in baseline prevalence (e.g., 20-24 vs 25-29 years; but chosen for subgroup consideration based on possibility of differing attendance at health care provider offices) may be combined. We will also consider providing estimates based on general population-level prevalence versus that estimated for high-risk individuals.

When event rates are less than 1%, the Peto odds ratio method will be used. However, when control groups are of unequal sizes, when large magnitude of effect is observed, or when events become more frequent (5%–10%), the Mantel-Haenszel method without correction factor will be used for quantitative synthesis [72]. Findings on relative effects from studies where no events occurred in either group will be qualitatively summarized; the data will be used for estimating a control event rate for estimation of absolute effects [73].

The decision to pool studies will not be based on the statistical heterogeneity; the I^2^ statistic (indicating heterogeneity rather than sampling error) and p values for heterogeneity will be reported but is recognized that the I^2^ is influenced by the number of studies and magnitude and direction of effects [73]. Rather, we will rely on interpretations of the clinical (related to our PICOTS) and methodological differences between studies.

For findings related to KQ 2, in addition to using studies directly comparing different screening approaches, we will consider using the results of indirect comparisons made between studies used for KQ 1 that differ in their screening programs by our intervention factors of interest but are similar in their “no screening” control group. We will first undertake qualitative assessment by plotting the results from the groups of studies and comparing the direction, magnitude, and 95% CIs of the effects sizes [72]. If comparable effectiveness is not plausible (e.g., 95% CIs do not overlap moderately), we will consider formal analytical approaches available such as indirect comparison meta-analysis (e.g., Bucher method) [74] or network meta-analysis (i.e., combining direct and indirect comparisons) [75, 76].

We will not directly combine results from trials with observational studies. Observational studies are generally considered to be of higher risks for bias, particularly with respect to selection biases (i.e., preferential screening based on perceptions of risk) making it more likely that groups will be dissimilar at baseline for known, or possibly unknown, confounders; commonly undertaken without a reported protocol, there is also more concern about reporting bias [77].

When a meta-analysis is not appropriate a narrative synthesis with accompanying tables and/or figures to present the data will be performed.

#### Sensitivity analysis

When substantial heterogeneity is suspected (i.e., it appears to impact the direction or magnitude of an effect in a clinically meaningful manner), we will conduct sensitivity analyses if appropriate (e.g., findings based only on low ROB studies (i.e.., all domains are assessed to have low ROB), studies screening for CT and/or NG with other STIs, inclusion of abstracts or other non-peer-reviewed outcome data as primary published data source, data requiring computation, analysis by invitation to screening rather than actual screened) or consider whether the heterogeneity is due to differing effects based on our population or intervention subgroups of interest (see Table 1 and section below).

#### Publication Bias

Where there are at least eight studies of varying size in a meta-analysis, we will analyze publication bias both visually using the funnel plot and quantitatively using Egger’s test [78].

#### Subgroup analyses

Our primary approach for evaluating the possibility of differential effects of screening for subgroups (see Tables 1 and 2) will be to record any within-study subgroup analyses performed by study investigators using individual patient data. Because these results are often based on diverse methodologies, may not align with our subgroup variables of interest, and can be difficult to interpret across the body of evidence, we will also perform our own subgroup analyses using study-level data, as possible, using formal statistical approaches (e.g., meta-regressions) or by stratifying the results of the pairwise meta-analyses by subgroup variables. When determining whether entire studies fall into a particular population subgroup category (e.g., high-risk), we will consider ≥80% of the study population meeting the criteria as sufficient. These analyses would rely on study-level data, such that the results would be considered observational in nature. We will test for evidence of subgroup effects quantitatively (significant at *p* = 0.05 although acknowledging that multiple subgroups may require lower *p* values for high certainty) [79], and also rely on available guidance when interpreting the credibility of the subgroup findings [65, 80].

#### Key question 3

Analysis for this KQ will be largely descriptive although will include narrative synthesis based on comparing and contrasting study findings by study methodology, populations, outcome presentations, and analysis. Additional patterns, with illustrative quotes or other information, may be drawn out from qualitative studies where suitable based on our variables and outcomes of interest. Findings based on differences between studies may also be created (e.g., if common or contrasting findings across studies generate unique patterns). We will report qualitative findings alongside quantitative findings when appropriate (e.g., both indicating relative preference for one outcome compared with another) or to help describe quantitative findings (e.g., why people may have chosen a particular outcome as most/least important). Only findings related to the KQ 1 and 2 primary outcomes will be extracted from each study.

### Assessment of the overall quality of the evidence using GRADE

Two reviewers experienced with the Grading of Recommendation, Assessment, Development and Evaluation (GRADE) approach will independently assess the quality of the body of evidence (our confidence that the effect estimate *is correct*) for each primary outcome of interest using the GRADE methodology for systematic review authors [60, 80–84]. Discrepancies will be resolved through discussion or another reviewer to reach consensus.

We will undertake separate GRADE assessments for experimental and observational study designs. Thereafter, we will give plausible reasons for any differences, and note pertinent limitations in both bodies of evidence; if we choose to combine the results into one overall quality grade, we will provide rationale.

Assessments will be entered into the GRADEPro software (https://gradepro.org/) and summarized in GRADE evidence profiles and Summary of Findings tables [85], in order for these to be used by the CTFPHC in an Evidence-to-Decision Table. Footnotes to the tables will explain all decisions to down- or upgrade the evidence, and will be organized by outcome. The CTFPHC will then use this evidence on each outcome, to assess the net balance of consequences, e.g., benefits and harms (depending on direction of effect for each outcome) of each option, patient preferences and values, and other elements of the GRADE methodology (feasibility, acceptability, costs, equity) to develop the recommendations on screening for chlamydia and for gonorrhea.

The CTFPHC may consider revising our conclusions about the GRADE quality assessment domains, based on whether or not the findings provide sufficient confidence in an estimate of the effect that is *adequate to support a particular recommendation* [60].

### Protocol amendments

Protocol amendments, including their description and date and timing within review conduct, will be documented in PROSPERO upon review completion. We will report on any changes to the protocol within the final manuscript.

## Discussion

The results section of the review will include a description of all studies, results of all analyses, including planned subgroup and sensitivity analyses, and evidence profiles and summary of findings tables incorporating assessment based on GRADE methods to communicate our confidence in the estimates of effect. In the discussion, we will summarize the main findings and their implications, compare our findings to others, and discuss limitations of the review and the available literature.

## Additional files

Additional file 1: Risk Indicators and Factors. (DOCX 24 kb)

Additional file 2: Components of a Screening Program. (DOCX 26 kb)

Additional file 3: Rationale and Scope of Guideline. (DOCX 24 kb)

Additional file 4: Recent National Guidance from Other Countries. (DOCX 19 kb)

Additional file 5: Interpreting Evidence Comparing Universal Versus Risk-Based Screening Strategies. (DOCX 149 kb)

Additional file 6: Methods for Integrating Existing Systematic Reviews into New Reviews. (DOCX 25 kb)

Additional file 7: Search Strategies. (DOCX 57 kb)

## Abbreviations

AE: Adverse effect
ARR: Absolute risk reduction
CI: Confidence interval
CT: Chlamydia trachomatis
CTFPHC: Canadian Task Force on Preventive Health Care
ERSC: Evidence Review and Synthesis Centre
GRADE: Grading of Recommendations Assessment, Development and Evaluation
HIV: Human immunodeficiency virus
ICC: Intraclass correlation coefficient
KQ: Key question
MD: Mean difference
MSM: Men who have sex with men
NAAT: Nucleic acid amplification tests
NG: Neisseria gonorrhoeae
NNS: Number needed to screen
PHAC: Public Health Agency of Canada
PICOTS: Population, interventions, comparators, outcomes, timing, setting
PID: Pelvic inflammatory disease
RCT: Randomized controlled trial
ROB: Risk of bias
RR: Relative risk
SD: Standard deviation
SMD: Standardized mean difference
STI: Sexually transmitted infection
USPSTF: U.S. Preventive Service Task Force
