# Supplementary material for: Screening for chlamydia and/or gonorrhea in primary health care: protocol for systematic review
Source: Syst Rev. 2018 Dec 26;7:248. doi: 10.1186/s13643-018-0904-5 (PMC6307186; doi:10.1186/s13643-018-0904-5)
Supplement: Supplementary file 1 — Risk Indicators and Factors. (DOCX 24 kb) [file 13643_2018_904_MOESM1_ESM.docx]

**Additional File 1**

**Risk Indicators and Factors**

**Sex and Age**

International population-based studies report that the rates of CT and NG infection in males and females may not differ substantially.[1-5] Reported cases of CT in Canada, however, are much more common in females which in part reflects the higher testing rate in this sex. In Canada, in 2014 the number of reported cases of CT among females was 1.7 times that of males.[6] This pattern is most apparent among females in the 15-19 (3.8 times male rates) and 20-24 (1.9 times male rate) year age groups, although it reverses once females reach 40 years of age (0.8 times male rate in 40-59 years). The rates of reported cases per 100,000 in persons of both sexes aged 15 to 29 years (females: 4,898; males: 2,360) comprised nearly 80% of all CT cases reported in 2014. Importantly, however, reported infections with CT have approximately doubled since 2005 in persons aged 30-39 (increased from 171 to 354 in females and from 168 to 321 in males, both per 100,000) and among those aged 40-59 years (increased from 23 to 57 in females and 36 to 73 in males, both per 100,000). Rates per 100,000 among those aged 10-14 (females: 58.7; males: 3.4) and aged 40-59 (females: 57.2; males: 72.8) are similar, with the exception of adolescent males. Reported cases in the 10-14 age category may underestimate prevalence due to low rates of testing in this age group. Among persons aged 60 and older, rates of reported cases of CT are low (females: 3; males: 9, both per 100,000) but increasing.[6]

Overall reported cases of NG in 2014 were about one-tenth of those for CT, and rates of reported cases per 100,000 were highest in the 20-24 age group (females: 161; males 198), followed by the 25-29 age group for males (190 per 100,000) and 15-19 age group for females (135 per 100,000).[6] In people less than 20 years of age, rates of NG cases were higher among females than males (1.9 times in 15-19 years), whereas among adults aged 20 and older, rates were higher for males (e.g., 1.2 times for 20-24 years, 2.1 times for 25-29 years, 2.8 times for 30-39 years). Case reporting of NG is increasing in most age groups, although the highest relative increase between 2005 and 2014 was among females aged 40-59 years (increase in rate from 2.6 to 8.3 per 100,000), followed by those aged 30-39 (from 15.2 to 38.1 per 100,000). In males, the highest relative increase between 2005 and 2014 was among those aged 25-29 years (99.7 to 190.5 per 100,000). Large rate increases were seen for people of both sexes in the 30-39 year (females: 15 to 38; males: 63 to 106) and 40-59 year (females: 2.6 to 8.3; males: 23 to 39) age groups. Rates are low (under 6 per 100,000) among those aged 10-14 and 60 and above.

**Geography**

The distribution of reported cases of CT varies geographically across Canada, with the highest rates (per 100,000) in Nunavut (3,510) and the Northwest Territories (1,894), followed by the Yukon (575), Saskatchewan (516), and Alberta (403), with the remainder of provinces being under 300 cases.[6] In 2014, the rate of reported cases of NG was significantly higher in Nunavut (891.1 per 100,000) than in other jurisdictions. Rates exceeding the national average of 45.8 per 100,000 were also observed in Alberta (46.2 per 100,000), Saskatchewan (110.2 per 100,000), Manitoba (86.4 per 100,000), Yukon (134.2 per 100,000) and the Northwest Territories (561.6 per 100,000).[6] Variation across Canada likely reflects differential risks for infection in relation to geography, in addition to other social determinants of health such as socioeconomic status, ethnicity, and other points of social vulnerability.

**Vulnerable Groups**

Prevalence of CT in young people in North America, Europe and Australia may be higher in those with lower socioeconomic status, particularly when using duration of education as the indicator, although results are inconsistent.[3, 4] Country of origin may play a role in Canadian prevalence data, with reviews of global prevalence finding highest rates of CT in those living in countries classified by the WHO as being in the Region of the Americas and Western Pacific Region, and for NG in Africa and the Western Pacific Region.[5] Migrant status may place one at higher risk due to an association with sexual abuse, abuse at work, language barriers and lack of social support networks.[7] In terms of subgroups of migrants, three studies of sex workers in high-income countries did not find increases in CT and NG infections in migrant versus non-migrant sex workers, as hypothesized and found for those in low-income countries; the weighted averages for CT was actually lower for migrants versus non-migrants (5% vs. 7%; OR 0.7, 95% CI 0.4-0.9).[8] Indigenous populations are also disproportionally affected due to multiple factors including but not limited to social context, including geography, history/colonialism, and demographics (young median age). A systematic review[9] on CT prevalence included two Canadian population-based studies in remote communities where Inuit participants had CT rates of 12% (Iqaluit, Nunavut)[10] and 3.7% (Nunavik, northern Quebec).[11] A systematic review of three studies in Australia found high prevalence rates of CT in Indigenous females (all ages 11% vs. <25 years 22%) and males (all ages 9% vs <25 years 15%).[12]

**Sexual Behaviours and Related Risk Factors**

Sexual behaviours considered to place one at high risk for infection include, but may not be limited to, inconsistent condom use, multiple sexual partners, partner(s) having concurrent partners, having new sexual partners, engaging in transactional sex, having sexual intercourse while under the influence of illicit drugs, and having recent sex with an infected person. The 2013/2014 Canadian Community Health Survey showed that 57.5% of sexually active Canadians aged 15 to 49, with two or more partners in the past year, reported using a condom the last time they had sex, but that use decreased with age.[13] While 70.8% of 15- to 19-year-olds used condoms, that number dropped to 58% for those 20 to 29. Findings from a large cross-sectional study [14] using a population representative sample in Quebec suggest condom use may not be that high; 70% and almost 20% of sexually active women aged 17-29 reported hormonal contraception or coitus interruptus, respectively, as their primary method of birth control, with less than a quarter using dual contraception (“pill” and condom) and many abandoning condoms when with a “partner”. Moreover, one in twenty young adults had their first consenting oral, vaginal or anal sex before the age of 14; before the age of 17, 50% of young women and 40% of young men had their first sexual encounter. Other studies have shown some concern about people on pre-exposure prophylaxis (PrEP) showing risk compensation, whereby they exhibit greater sexual risk after adoption of the safety measure.[15]

Risk factors related to sexual behaviours are often reported indirectly based on group membership rather than actual behaviours. For example, incarcerated people have a relatively high prevalence of CT and NG infections (CT: females 12%, males 6%; NG: females 6%, males 1.4%).[16] Prevalence rates of CT in correctional facilities in the United States are high in males (adults 3-8%; adolescents 3-9%) and females (adults 6-15%; adolescents 5-25%); rates were low (<2%) for NG in adult and adolescent males but higher in females (adults 2.5-3.4%; adolescents 3-7%).[17] MSM are disproportionally affected, particularly for NG and extragenital infection. Population groups considered at high-risk due to behavioural factors may also be disproportionally exposed to social factors. For example, as recognized by the World Health Organization, MSM and transgender people experience significant barriers to quality health care because of stigma against homosexuality and ignorance about gender variance in mainstream society.^35^ While underlying correlates of STI risk, as well as specific sexual health needs of transgender people are distinct from those of MSM, both groups may delay or avoid seeking STI-related information, care and services as a result of anticipated homophobia, transphobia, ignorance and insensitivity.[18]

**Biological and Epidemiological Factors**

Individuals infected with NG may be (possibly up to 20-40%) co-infected with CT, although fewer people with CT also have NG.[19, 20] Having a concurrent human immunodeficiency virus (HIV) infection or other STI is also seen as a risk factor due to epidemiological and biological factors. Higher risk for STIs in younger age groups can be attributed to behavioural (e.g., condomless sex) but also biological factors (e.g., cervical ectopy in adolescent females [i.e., glandular/columnar epithelial cells are present on outer surface rather than only inside of cervical canal]).[21]

**Additional File 1 References**

1. Dielissen PW, Teunissen DA, Lagro-Janssen AL. Chlamydia prevalence in the general population: is there a sex difference? A systematic review. BMC Infect Dis. 2013;13:534.

2. Redmond SM, Alexander-Kisslig K, Woodhall SC, van den Broek IV, van Bergen J, Ward H, Uuskula A, Herrmann B, Andersen B, Gotz HM, et al. Genital chlamydia prevalence in Europe and non-European high income countries: systematic review and meta-analysis. PLoS ONE. 2015;10:e0115753.

3. Sheringham J, Mann S, Simms I, Stafford M, Hart GJ, Raine R. It matters what you measure: a systematic literature review examining whether young people in poorer socioeconomic circumstances are more at risk of chlamydia. Sex Transm Infect. 2013;89:175-180.

4. Crichton J, Hickman M, Campbell R, Batista-Ferrer H, Macleod J. Socioeconomic factors and other sources of variation in the prevalence of genital chlamydia infections: a systematic review and meta-analysis. BMC Public Health. 2015;15:729.

5. Newman L, Rowley J, Vander Hoorn S, Wijesooriya NS, Unemo M, Low N, Stevens G, Gottlieb S, Kiarie J, Temmerman M. Global estimates of the prevalence and incidence of four curable sexually transmitted infections in 2012 based on systematic review and global reporting. PLoS ONE. 2015;10:e0143304.

6. Public Health Agency of Canada. Report on Sexually Transmitted Infections in Canada: 2013-2014. Ottawa, ON: Centre for Communicable Diseases and Infection Control, Infectious Disease Prevention and Control Branch, PHAC. 2017. <https://www.canada.ca/en/public-health/services/publications/diseases-conditions/report-sexually-transmitted-infections-canada-2013-14.html> Accessed 22 April 2018.

7. Ochoa SC, Sampalis J. Risk perception and vulnerability to STIs and HIV/AIDS among immigrant Latin-American women in Canada. Cul Health Sex. 2014;16:412-425.

8. Platt L, Grenfell P, Fletcher A, Sorhaindo A, Jolley E, Rhodes T, Bonell C. Systematic review examining differences in HIV, sexually transmitted infections and health-related harms between migrant and non-migrant female sex workers. Sex Transm Infect. 2013;89:311-19.

9. European Centre for Disease Prevention and Control. Chlamydia control in Europe: literature review. Stockholm: ECDC. 2014. <https://ecdc.europa.eu/en/publications-data/chlamydia-control-europe-literature-review> Accessed 22 April 2018.

10. Steenbeek A, Tyndall M, Sheps S, Rothenberg R. An epidemiological survey of chlamydial and gonococcal infections in a Canadian arctic community. Sex Transm Dis. 2009;36:79-83.

11. Hodgins S, Peeling RW, Dery S, Bernier F, LaBrecque A, Proulx JF, Joly J, Alary M, Mabey D. The value of mass screening for chlamydia control in high prevalence communities. Sex Transm Infect. 2002;78(Suppl 1):i64-8.

12. Lewis D, Newton DC, Guy RJ, Ali H, Chen MY, Fairley CK, Hocking JS. The prevalence of Chlamydia trachomatis infection in Australia: a systematic review and meta-analysis. BMC Infect Dis. 2012;12:113.

13. Statistics Canada. 2013/14 Canadian Community Health Survey. Table 7. Canadians who used a condom during last sexual intercourse, by age group and sex, household population aged 15 to 49, 2013/2014 Ottawa, ON; 2015.

14. Lambert G, Mathieu-Chartier S, Goggin P, Maurais E. Étude PIXEL – Portrait de la santé sexuelle des jeunes adultes au Québec. QuébecL Institut national de santè publique du Quèbec. 2014.

15. Beymer MR, DeVost MA, Weiss RE, Dierst-Davies R, Shover CL, Landovitz RJ, Beniasians C, Talan AJ, Flynn RP, Krysiak R, et al. Does HIV pre-exposure prophylaxis use lead to a higher incidence of sexually transmitted infections? A case-corssover study of men who have sex with men in Los Angeles, California. Sex Trans Inf. 2018.

16. Kouyoumdjian FG, Leto D, John S, Henein H, Bondy S. A systematic review and meta-analysis of the prevalence of chlamydia, gonorrhoea and syphilis in incarcerated persons. Int J STD AIDS. 2012;23:248-54.

17. Bernstein KT, Chow JM, Pathela P, Gift TL. Bacterial sexually transmitted disease screening outside the clinic--implications for the modern sexually transmitted disease program. Sex Transm Dis. 2016;43:S42-52.

18. WHO HIV/AIDS Programme. Prevention and treatment of HIV and other sexually transmitted infections among men who have sex with men and transgender people. Recommendations for a public health approach. 2011. <http://www.who.int/hiv/pub/guidelines/msm_guidelines2011/en/> Accessed 22 April 2018.

19. Creighton S, Tenant-Flowers M, Taylor CB, Miller R, Low N. Co-infection with gonorrhoea and chlamydia: how much is there and what does it mean? Int J STD AIDS. 2003;14:109-13.

20. McMillan A, Manavi K, Young H. Concurrent gonococcal and chlamydial infections among men attending a sexually transmitted diseases clinic. Int J STD AIDS. 2005;16:357-61.

21. Lee V, Tobin JM, Foley E. Relationship of cervical ectopy to chlamydia infection in young women. J Fam Plann Reprod Health Care. 2006;32:104-6.
