# Supplementary material for: Screening for chlamydia and/or gonorrhea in primary health care: protocol for systematic review
Source: Syst Rev. 2018 Dec 26;7:248. doi: 10.1186/s13643-018-0904-5 (PMC6307186; doi:10.1186/s13643-018-0904-5)
Supplement: Supplementary file 2 — Components of a Screening Program. (DOCX 26 kb) [file 13643_2018_904_MOESM2_ESM.docx]

**Additional File 2**

**Components of a Screening Program**

**Diagnostic Tests**

Nucleic acid amplification tests (NAATs) have become the diagnostic gold standard for detecting CT and NG because they are more (≥10-50%) sensitive than culture.[1, 2] NAATs are highly sensitive (86-98%) for both CT and NG at genital sites,[3] and have been the most widely used tests in high-income countries for genital infections since they were introduced in the mid-1990s. The sensitivity does not appear to differ based on the presence or absence of symptoms.[4] The specificity of NAATs is usually claimed to be very high (>99%) although some studies have shown lower specificities around 97%[2-5] or even 90% for male NG infection.[4] NAATs do not require viable organisms, and many can detect both CT and NG. The increased sensitivity of NAATs is attributable to their theoretic ability to produce a positive signal from as little as a single copy of the target DNA or RNA. NAATs can detect CT in first-void urine (i.e., first-catch [not mid-stream] with person ideally, but not necessarily, having last voided at least 2 hours previously) and vulvovaginal, oropharyngeal, or rectal swabs which makes them suitable for large volume screening. Vaginal swabs from females and urine samples from males appear to be the most accurate for self-collection,[6] although a review of studies by the U.S. Centers for Disease Control and Prevention on accuracy and acceptability of rectal swabs, self- or provider-collected, using NAATs found good evidence of their performance and acceptability.[7] Uncertainty was found for oropharyngeal and penile meatal NAAT swab performance. NAATs for NG in the pharynx may have lower specificity. Self-collection of a large variety of sample types has been shown to be highly acceptable.[8, 9]

Commonly used NAATs techniques include: polymerase chain reaction, strand displacement amplification, and transcription-mediated amplification.[4, 10] NAATs are not used universally across Canada for testing of rectal and pharyngeal swabs. They are not yet approved by Health Canada for these types of specimens, and laboratory validation for each specimen and infection type is required. If culture is used for these sites, the lower sensitivity (detecting 10-50% fewer cases than NAATs) and stability of organisms will need to be considered. Commercial home test kits are available in countries such as the United Kingdom and United States, although none are approved yet by Health Canada.

Although the accuracy of CT and NG NAAT tests is high, their predictive values will be affected by the prevalence of the infections in the tested population. For example, at any given specificity the rate of false positives will increase with declining prevalence. Moreover, small changes in the tests’ specificity may have a large impact on the number of patients receiving a false positive result as the prevalence drops. For example, if a test with a specificity and sensitivity of 99% and 85%, respectively, is used to screen a population of 10,000 patients with a CT prevalence of 10% (i.e., 1,000 patients have an infection), on average 940 tests will be positive: 850 patients with a positive result will actually be infected, and 90 will not be infected (i.e., 9.6% false-positives). The proportion of those with a positive result who are truly positive (positive predictive value [PPV]) is 850/940 = 90%. When this same test is used to screen 10,000 patients in a population with a CT prevalence of only 2% (i.e., 200 patients have an infection), an average of 268 test results will be positive: 170 patients will be infected and 98 will not. The PPV is 170/268 = 63% (i.e., false positive rate 37%). If the specificity of the test decreases to 97%, the PPV will reach <40% and false positive will approach 60% for a 2% population prevalence.[1, 2] Studies finding high (>90%) concordance rates of repeat tests prompted removal of earlier recommendations to retest positive cases in attempts to increase the PPV.[5]

**Treatment**

Canadian guidelines exist for the treatment of uncomplicated CT and NG with antibiotics.[1, 11] The treatment components of these guidelines are updated on a regular basis and, for NG, reflect on-going surveillance for the prevalence of antimicrobial resistance. CT is usually treated with azithromycin, a macrolide antibiotic) or doxycycline, a tetracycline, which have been shown to have similar efficacy for genital infections.[12, 13] Doxycycline may be more effective than azithromycin for rectal infections, but these findings are uncertain with upcoming trial data aiming to provide higher quality evidence.[14, 15] The 1 gram single-dose azithromycin regimen is preferable to the doxycycline 7-day course usually twice daily to overcome poor adherence. Re-administration is recommended upon vomiting, especially for the single-dose azithromycin. For a positive anogenital or pharyngeal NG result (culture or NAAT), first-line treatment includes a cephalosporin (either ceftriaxone 250 mg intramuscular [one-time dose] or oral cefixime 800 mg orally [one-time dose]) plus azithromycin 1 gram orally (one-time dose). Combination therapy using two antibiotics with different mechanisms of action strives to improve treatment efficacy as well as to potentially delay the emergence of cephalosporin-resistant NG. This regime will also target CT, which may be concomitant.[11] Quinolones are no longer recommended due to high antimicrobial resistance, unless a NG strain has been shown to be sensitive by culture. Culture for NG antibiotic sensitivities is recommended before treatment of NG in all symptomatic cases when empiric treatment was given, or for screened people before receiving the prescription to treat appropriately, as well as to monitor the prevalence of resistance,^91^ although culture may not be undertaken or available in some jurisdictions.

**Post-Treatment Follow-up**

Treatment of sexual partners is recommended, even if not tested, and expedited partner therapy strategies may reduce prevalence and index case reinfection.[16-18] Retesting is important to detect reinfection, although recommended timeframes (6 weeks to 12 months) differ depending on the organism, gender of the individual, treatment compliance and other factors.[1, 19-21] Routine test of cure (TOC) for CT is not usually recommended due to the high efficacy of recommended treatment regimens. In recommendations for CT of the Public Health Agency of Canada, TOC is recommended where compliance is suboptimal, if an alternative regimen is used, in prepubertal children, and in pregnant women. For NG, TOC is recommended in all cases, but in particular for similar situations as with CT as well as additional scenarios including but not limited to previous treatment failures (cases or partners) and suspected or known antimicrobial resistance.[1] If undertaken, TOC should be performed at least 2-3 weeks (NG) or 3-4 weeks (CT) after treatment when using NAAT testing because residual nucleic acids from dead organisms may persist, leading to false-positive tests in shorter follow-up periods.[7, 22-24] The longer timeframes used for retesting to detect reinfection will not easily separate between this outcome and treatment failure or non-adherence, or false positives.

Behavioural counselling[25-33] to avoid reinfection is recommended during follow up. Brief (including in-person, digital and video)[30] and more intensive counselling efforts may both be effective for reducing STI risk behaviors—particularly when tailored to the participants(s) and when including education (e.g., condom skills), skills development (e.g., communication and negotiation skills) and presentation of arguments to change attitudes[27, 33]—although findings for ultimately reducing STIs are variable.[31, 32]

**Additional File 2 References**

1. Pubic Health Agency of Canada. Canadian Guidelines on Sexually Transmitted Infections. 2010. <https://www.canada.ca/en/public-health/services/infectious-diseases/sexual-health-sexually-transmitted-infections/canadian-guidelines.html> Accessed 22 April 2018.

2. Johnson RE, Newhall WJ, Papp JR, Knapp JS, Black CM, Gift TL, Steece R, Markowitz LE, Devine OJ, Walsh CM. Screening tests to detect Chlamydia trachomatis and Neisseria gonorrhoeae infections--2002. MMWR Recomm Rep. 2002;51:1-38.

3. Zakher B, Cantor AG, Pappas M, Daeges M, Nelson HD: Screening for gonorrhea and Chlamydia: a systematic review for the U.S. Preventive Services Task Force. Ann Intern Med. 2014;161:884-93.

4. Cook RL, Hutchison SL, Ostergaard L, Braithwaite RS, Ness RB. Systematic review: noninvasive testing for Chlamydia trachomatis and Neisseria gonorrhoeae. Ann Intern Med. 2005;142:914-25.

5. Papp J, Schachter J, Gaydos CA, Van Der Pol B. Recommendations for the laboratory-based detection of Chlamydia trachomatis and Neisseria gonorrhoeae--2014. MMWR Recomm Rep. 2014;63:1-19.

6. Lunny C, Taylor D, Hoang L, Wong T, Gilbert M, Lester R, Krajden M, Ogilvie G. Self-collected versus clinician-collected sampling for chlamydia and gonorrhea screening: a systemic review and meta-analysis. PLoS ONE. 2015;10:e0132776.

7. Geisler WM. Diagnosis and management of uncomplicated Chlamydia trachomatis infections in adolescents and adults: summary of evidence reviewed for the 2015 Centers for Disease Control and Prevention Sexually Transmitted Diseases Treatment Guidelines. Clin Infect Dis. 2015;61(Suppl 8):S774-84.

8. Paudyal P, Llewellyn C, Lau J, Mahmud M, Smith H. Obtaining self-samples to diagnose curable sexually transmitted infections: a systematic review of patients' experiences. PLoS ONE. 2015;10:e0124310.

9. Odesanmi TY, Wasti SP, Odesanmi OS, Adegbola O, Oguntuase OO, Mahmood S. Comparative effectiveness and acceptability of home-based and clinic-based sampling methods for sexually transmissible infections screening in females aged 14-50 years: a systematic review and meta-analysis. Sex Health. 2013;10:559-69.

10. LeFevre ML. Screening for chlamydia and gonorrhea: U.S. Preventive Services Task Force recommendation statement. Ann Intern Med. 2014;161:902-10.

11. Pogany L, Romanowski B, Robinson J, Gale-Rowe M, Latham-Carmanico C, Weir C, Wong T. Management of gonococcal infection among adults and youth: new key recommendations. Can Fam Physician. 2015;61:869-73.

12. Lau CY, Qureshi AK. Azithromycin versus doxycycline for genital chlamydial infections: a meta-analysis of randomized clinical trials. Sex Transm Dis. 2002;29:497-502.

13. Kong FY, Tabrizi SN, Law M, Vodstrcil LA, Chen M, Fairley CK, Guy R, Bradshaw C, Hocking JS. Azithromycin versus doxycycline for the treatment of genital chlamydia infection: a meta-analysis of randomized controlled trials. Clin Infect Dis. 2014;59:193-205.

14. Kong FY, Tabrizi SN, Fairley CK, Vodstrcil LA, Huston WM, Chen M, Bradshaw C, Hocking JS. The efficacy of azithromycin and doxycycline for the treatment of rectal chlamydia infection: a systematic review and meta-analysis. J Antimicrob Chemother. 2015;70:1290-7.

15. Lau A, Kong F, Fairley CK, Donovan B, Chen M, Bradshaw C, Boyd M, Amin J, Timms P, Tabrizi S, Regan DG, Lewis DA, McNulty A, Hocking JS. Treatment efficacy of azithromycin 1 g single dose versus doxycycline 100 mg twice daily for 7 days for the treatment of rectal chlamydia among men who have sex with men – a double-blind randomised controlled trial protocol. BMC Infect Dis. 2017;17:35.

16. Althaus CL, Turner KM, Mercer CH, Auguste P, Roberts TE, Bell G, Herzog SA, Cassell JA, Edmunds WJ, White PJ, Ward H, Low N. Effectiveness and cost-effectiveness of traditional and new partner notification technologies for curable sexually transmitted infections: observational study, systematic reviews and mathematical modelling. Health Technol Assess. 2014;18:1-100.

17. Hogben M, Collins D, Hoots B, O'Connor K. Partner services in sexually transmitted disease prevention programs: a review. Sex Transm Dis. 2016;43:S53-62.

18. Schillinger JA, Gorwitz R, Rietmeijer C, Golden MR. The expedited partner therapy continuum: a conceptual framework to guide programmatic efforts to increase partner treatment. Sex Transm Dis. 2016;43:S63-75.

19. Public Health England: Chlamydia screening: evidence summary and briefing. 2014. <https://www.gov.uk/government/publications/chlamydia-screening-evidence-summary-and-briefing> Accessed 23 April 2018.

20. van der Helm JJ, Koekenbier RH, van Rooijen MS, Schim van der Loeff MF, de Vries HJC. What is the optimal time to retest patients with a urogenital chlamydia infection? A randomized controlled trial. Sex Transm Dis. 2018;45:132-7.

21. Scottish Intercollegiate Guidelines Network: Management of genital Chlamydia trachomatis infection: a national clinical guideline 2009. [www.sign.ac.uk/assets/sign109.pdf Accessed 23 April 2018](http://www.sign.ac.uk/assets/sign109.pdf%20Accessed%2023%20April%202018).

22. Dukers-Muijrers NH, Morre SA, Speksnijder A, van der Sande MA, Hoebe CJ. Chlamydia trachomatis test-of-cure cannot be based on a single highly sensitive laboratory test taken at least 3 weeks after treatment. PLoS ONE. 2012;7:e34108.

23. Workowski KA, Bolan GA. Sexually transmitted diseases treatment guidelines, 2015. MMWR Recomm Rep. 2015;64:1-137.

24. Nwokolo NC, Dragovic B, Patel S, Tong CY, Barker G, Radcliffe K. 2015 UK national guideline for the management of infection with Chlamydia trachomatis. Int J STD AIDS. 2016;27:251-67.

25. Brookmeyer KA, Hogben M, Kinsey J. The role of behavioral counseling in sexually transmitted disease prevention program settings. Sex Transm Dis. 2016;43:S102-12.

26. Cooper B, Toskin I, Kulier R, Allen T, Hawkes S. Brief sexuality communication--a behavioural intervention to advance sexually transmitted infection/HIV prevention: a systematic review. BJOG. 2014;121(Suppl 5):92-103.

27. Covey J, Rosenthal-Stott HE, Howell SJ. A synthesis of meta-analytic evidence of behavioral interventions to reduce HIV/STIs. J Behavior Med. 2016;39:371-85.

28. Eaton LA, Huedo-Medina TB, Kalichman SC, Pellowski JA, Sagherian MJ, Warren M, Popat AR, Johnson BT. Meta-analysis of single-session behavioral interventions to prevent sexually transmitted infections: implications for bundling prevention packages. Am J Public Health. 2012;102:e34-44.

29. Goesling B, Colman S, Trenholm C, Terzian M, Moore K. Programs to reduce teen pregnancy, sexually transmitted infections, and associated sexual risk behaviors: a systematic review. J Adolesc Health. 2014;54:499-507.

30. Long L, Abraham C, Paquette R, Shahmanesh M, Llewellyn C, Townsend A, Gilson R. Brief interventions to prevent sexually transmitted infections suitable for in-service use: a systematic review. Prevent Med. 2016;91:364-82.

31. Macaya Pascual A, Ferreres Riera JR, Campoy Sanchez A. Behavioral interventions for preventing sexually transmitted infections and unintended pregnancies: an overview of systematic reviews. Actas Dermo-Sifiliograficas. 2016;107:301-17.

32. O'Connor EA, Lin JS, Burda BU, Henderson JT, Walsh ES, Whitlock EP. Behavioral sexual risk-reduction counseling in primary care to prevent sexually transmitted infections: a systematic review for the U.S. Preventive Services Task Force. Annal Inter Med. 2014;161:874-83.

33. Petrova D, Garcia-Retamero R. Effective evidence-based programs for preventing sexually-transmitted infections: a meta-analysis. Curr HIV Res. 2015;13:432-38.
