# Supplementary material for: Screening for chlamydia and/or gonorrhea in primary health care: protocol for systematic review
Source: Syst Rev. 2018 Dec 26;7:248. doi: 10.1186/s13643-018-0904-5 (PMC6307186; doi:10.1186/s13643-018-0904-5)
Supplement: Supplementary file 3 — Rationale and Scope of Guideline. (DOCX 24 kb) [file 13643_2018_904_MOESM3_ESM.docx]

**Additional File 3**

**Rationale and Scope of Guideline**

Several factors provide a rationale for the CTFPHC to issue recommendations for CT and NG screening. The Public Health Agency of Canada (PHAC) produces guidance to assist primary care and public health professionals in the diagnosis, treatment, and management of the most common STIs.[1] PHAC’s 2010 Chlamydial Infections chapter lists risk factors for infection (e.g., previous STI or sexual contact with CT-infected person, new sexual partner or more than two partners in the past year, vulnerable populations [e.g., sex trade workers, injection drug users, street youth]), and recommends screening of those at-risk groups, in addition to all sexually active females and males under 25 years of age and pregnant women. While the intent was to screen at-risk groups of any age (personal communication by Dr. Margaret Gale-Rowe of PHAC Professional Guidelines and Public Health Practice Division), it appears that this distinction is not clear. For example, Public Health Ontario has interpreted the recommendation to be limited to males and females <25 years of age. Further, a review of recent studies on CT testing in the province of Ontario revealed that clinicians interpret the recommendations to be exclusive to the under-25 age population without including other older high-risk groups.[2, 3] Further rationale for updated guidance comes from epidemiological changes in reported rates of CT in Canada. The PHAC 24-year age limit is aligned with 2004 Canadian statistics for which the highest reported cases of CT were among those aged 15-24 years. However, the number or reported cases for those aged 25-29 has been increasing ever since and, in 2014, surpassed the rates in 2004/5 for 15-19 year olds.[4] Furthermore, even with a lack of effectiveness data from screening for CT in males, PHAC advised screening males given that they serve as an often overlooked source for infections and reinfections of their female partners.

For NG, PHAC guidance describes similar risk factors as for CT but specific attention is additionally given to males who have condomless sex with males. Case finding and partner notification are stated as critical for controlling NG, but specific methods for case finding are not described and could be interpreted as screening (personal communication with previous Chair of the Canadian STI Guidelines, Dr. Tom Wong, clarified that case finding is meant to indicate active seeking of signs and symptoms in at-risk individuals rather than screening asymptomatic individuals).

Neither PHAC recommendation was based on a systematic review of the evidence on screening. Further, most provincial guidelines focus on diagnostic tests and treatment without providing specific recommendations about screening.[5] An exception is the province of Quebec’s guideline which recommends the annual (or more frequent) evaluation of risk factors and screening based on the clinical judgment of a physician, nurse, or nurse practitioner for the following high risk groups: 1) all sexually active individuals ≤25 years without other risk factors for CT, all females ≤25 years for NG and males ≤25 years living in the James Bay Cree or Nunavik Territories for NG; 2) individuals with identified risk factors (i.e., females with a new sexual partner or more than one concurrent sexual partner for CT and NG, males for CT only; individuals with ‘anonymous’ sexual partners (i.e., met at a bar, club, or on the Internet for the purpose of sexual relations) or more than three sexual partners in the past year, emigration from a region where bacterial STIs are endemic, MSM, sex workers and their clients, individuals who test positive for another STI, sexual partners with risk factors or who test positive for CT or NG). Retesting for infection is recommended after 3 to 6 months, including re-screening for CT in the presence of NG due to high co-infection rates. The guideline provides recommendations for testing methods that may be used (anatomical site, e.g., pharyngeal and/or anus/rectum if indicated), and partner notification. [6] Apart from Quebec’s guidance, other screening recommendation statements are not specific for different screening settings and approaches, screening intervals, or testing methods (e.g., personnel, anatomical site).

Apart from the abovementioned rationale for CTFPHC guidance, there is the potential need to consider impacts on infection prevalence and/or screening implementation in relation to changes in guidance for cervical cancer screening. Two recent observational studies in Ontario have found reductions in STI and CT screening after recommendations to delay cervical cancer screening until age 21 (Cancer Care Ontario in May 2012) or age 25 (CTFPHC in January 2013).[2, 3] A retrospective before-after study of 200 women aged 19-25 years found a 50% reduction (from 40% [before May 2012] to 20% [after November 2012]; odds ratio [OR] 0.38, 95% confidence interval [CI] 0.19 to 0.74) of STI screening in family practice units in Toronto.[2] Another study using an interrupted time series design (4 quarters before and after May 2012) with population-based physician billing claims and public health surveillance data in Ontario found reductions in CT testing for females in all ages 15-29, but the largest decrease for those aged 15-19 (25.5% relative reduction [RR], 95% CI -32.7 to -18.2).[3] Testing for CT in males aged 20-29 years increased to a small extent (ages 20-24: RR 7.1%, 95% CI 1.2 to 12.9 ; ages 25-29: RR 10.5%, 95% CI 4.1 to 16.9), which may be due to increased availability of urine tests. Confounding factors, such as possible changes in sexually activity or other risk factors, cannot be completely accounted for in these studies, and the effects on population prevalence over the longer term are difficult to predict.

The guideline that this evidence review will support will not focus on screening for CT or NG during pregnancy, nor will it focus on screening for other STIs, or primary prevention of STIs. CT and NG will be the focus of this guideline as they represent the first and second most prevalent STIs in Canada. Furthermore, co-infection with CT is fairly common in people with NG and clinicians may screen for these infections simultaneously. This practice is facilitated by the availability of laboratory tests that can evaluate both organisms from a single sample and test, and because first-line treatment for NG can in most uncomplicated cases also treat CT, even though the effectiveness of screening for both in all cases has not been evaluated. Systematic reviews are available related to prevention of STIs, including behavioural counselling or other primary care interventions to encourage risk reduction.[7-14] Screening during pregnancy will not be included, owing to available resources and associated scope of work (e.g., different outcomes and testing and treatment considerations for mothers and newborns), as well as awareness of upcoming national guidance based on systematic reviews, for which the CTFPHC could adapt or appraise in the future. For CT, the scope of this guideline includes screening for serovars (strains) D-K, but not for serovars L1-3 which cause *lymphogranuloma venereum* (LGV). LGV is primarily an infection of the lymphatics and lymph nodes, and although it is increasing in some populations (i.e., men who have sex with men [MSM]) it is still rare in Canada (104 confirmed cases between 2004 and 2012).[15] It should be recognized, though, that most tests for CT will identify but not distinguish non-LGV and LGV serovars;[16] additional testing is required to differentiate between these serovars.

The most directly relevant screening approaches for this CTFPHC guideline are those delivered by primary health care providers, where participants are identified for screening via attendance at a clinic, more systematic means (e.g., mailed invitation via health register) or some other form of invitation, from locations considered a first point of contact with the health system such as clinician offices (e.g., family physician, pediatrician, nurse practitioner) and community health settings (e.g., school health clinics, emergency departments, sexually transmitted infection [STI] clinics, out-patient clinics, pharmacies, prisons, substance use clinics, family planning/fertility/abortion clinics, public health clinics). Evidence from screening undertaken in specialist settings (e.g., inpatient units, obstetrics/gynecology offices, infectious disease clinics), via outreach programming (e.g., sex venues, sports facilities, online), or using regional population register-based approaches (e.g., postal kits delivered to homes, not directly related to primary health care) will be included in this review but viewed as less direct evidence. This is primarily because health care providers of screening are the target audience of CTFPHC recommendations; there may also be differences in feasible approaches and in the prevalence of infections in [higher risk] populations targeted by outreach approaches,[17]) both of which would influence interpretations of the effects of screening in primary health care. This aligns with Health Canada’s functional definition of primary health care: (i) to provide first-contact services; and (ii) to coordinate care to ensure continuity and ease of movement across the system, so that care remains integrated (http://healthycanadians.gc.ca/health-system-systeme-sante/services/primary-primaires/about-apropos-eng.php).

**Additional File 3 References**

1. Pubic Health Agency of Canada. Canadian Guidelines on Sexually Transmitted Infections. 2010. <https://www.canada.ca/en/public-health/services/infectious-diseases/sexual-health-sexually-transmitted-infections/canadian-guidelines.html> Accessed 22 April 2018.

2. Bogler T, Farber A, Stall N, Wijayasinghe S, Slater M, Guiang C, Glazier RH. Missed connections: Unintended consequences of updated cervical cancer screening guidelines on screening rates for sexually transmitted infections. Can Fam Phys. 2015;61:e459-6.

3. Naimer MS, Kwong JC, Bhatia D, Moineddin R, Whelan M, Campitelli MA, Macdonald L, Lofters A, Tuite A, Bogler T, et al. The effect of changes in cervical cancer screening guidelines on chlamydia testing. Ann Fam Med. 2017;15:329-34.

4. Public Health Agency of Canada. Report on Sexually Transmitted Infections in Canada: 2013-2014. Ottawa, ON: Centre for Communicable Diseases and Infection Control, Infectious Disease Prevention and Control Branch, PHAC. 2017. <https://www.canada.ca/en/public-health/services/publications/diseases-conditions/report-sexually-transmitted-infections-canada-2013-14.html> Accessed 22 April 2018.

5. Public Health Agency of Canada. Canadian Guidelines on Sexually Transmitted Infections: Provincial/Territorial Communicable Disease Guidleines. 2017. <http://www.phac-aspc.gc.ca/std-mts/sti-its/pt-sti-its-eng.php> Accessed 22 April 2018.

6. Infections Transmissible Sexuellement et Par le Sang (ITSS). Guide Quebecois de Depistage. Québec: Institut national de santé publique du Québec; 2017.

7. Cooper B, Toskin I, Kulier R, Allen T, Hawkes S. Brief sexuality communication--a behavioural intervention to advance sexually transmitted infection/HIV prevention: a systematic review. BJOG. 2014;121(Suppl 5):92-103.

8. Goesling B, Colman S, Trenholm C, Terzian M, Moore K. Programs to reduce teen pregnancy, sexually transmitted infections, and associated sexual risk behaviors: a systematic review. J Adolesc Health. 2014;54:499-507.

9. O'Connor EA, Lin JS, Burda BU, Henderson JT, Walsh ES, Whitlock EP. Behavioral sexual risk-reduction counseling in primary care to prevent sexually transmitted infections: a systematic review for the U.S. Preventive Services Task Force. Annal Inter Med. 2014;161:874-83.

10. Petrova D, Garcia-Retamero R. Effective evidence-based programs for preventing sexually-transmitted infections: a meta-analysis. Curr HIV Res. 2015;13:432-38.

11. Brookmeyer KA, Hogben M, Kinsey J. The role of behavioral counseling in sexually transmitted disease prevention program settings. Sex Transm Dis. 2016;43:S102-12.

12. Covey J, Rosenthal-Stott HE, Howell SJ. A synthesis of meta-analytic evidence of behavioral interventions to reduce HIV/STIs. J Behavior Med. 2016;39:371-85.

13. Long L, Abraham C, Paquette R, Shahmanesh M, Llewellyn C, Townsend A, Gilson R. Brief interventions to prevent sexually transmitted infections suitable for in-service use: a systematic review. Prevent Med. 2016;91:364-82.

14. Macaya Pascual A, Ferreres Riera JR, Campoy Sanchez A. Behavioral interventions for preventing sexually transmitted infections and unintended pregnancies: an overview of systematic reviews. Actas Dermo-Sifiliograficas. 2016;107:301-17.

15. Totten S MR, Payne E, et al. Chlamydia and lymphogranuloma venereum in Canada: 2003-2012 Summary Report. Canada Communicable Disease Report (CCDR). 2015. <https://www.canada.ca/en/public-health/services/reports-publications/canada-communicable-disease-report-ccdr/monthly-issue/2015-41/ccdr-volume-41-02-february-5-2015/ccdr-volume-41-02-february-5-2015.html> Accessed 22 April 2018.

16. Association of Public Health Laboratories and Centers for Disease Control and Prevention (CDC). Laboratory Diagnostic Testing for Chlamydia trachomatis and Neisseria gonorrhoeae: Expert Consultation Meeting Summary Report. CDC. 2009.

17. Hengel B, Jamil MS, Mein JK, Maher L, Kaldor JM, Guy RJ. Outreach for chlamydia and gonorrhoea screening: a systematic review of strategies and outcomes. BMC Public Health. 2013;13:1040.
