# Supplementary material for: Screening for chlamydia and/or gonorrhea in primary health care: protocol for systematic review
Source: Syst Rev. 2018 Dec 26;7:248. doi: 10.1186/s13643-018-0904-5 (PMC6307186; doi:10.1186/s13643-018-0904-5)
Supplement: Supplementary file 4 — Recent National Guidance from Other Countries. (DOCX 19 kb) [file 13643_2018_904_MOESM4_ESM.docx]

**Additional File 4**

**Recent National Guidance from Other Countries**

The United States Preventive Services Task Force (USPSTF) recently released updated guidance on screening for CT and NG in adolescents and adults, including pregnant women.[1] They recommend screening for both CT and NG in sexually active females aged 24 years or younger and in older women who are at increased risk for infection. These were “B” recommendations, meaning “The USPSTF recommends the service. There is high certainty that the net benefit is moderate or there is moderate certainty that the net benefit is moderate to substantial.” The statement for CT was based on screening trial evidence from which the USPSTF considered the benefits to be moderate in magnitude, while that for NG was based on “the large proportion of cases that are asymptomatic, the effectiveness of antibiotic treatment to reduce infections, and the high morbidity associated with untreated infections.” For men, the current evidence was considered insufficient to assess the balance of benefits and harms of screening for CT and NG (Insufficient evidence, I level statement). These statements are inclusive of pregnant women, but the USPSTF did not make a separate statement for screening timing or intervals during pregnancy.

In the United Kingdom, national guidelines for the testing and treatment of CT (2015) and NG (2011) infections have been produced by the British Association of Sexual Health and HIV, but neither recommendation specifies who, when nor how to offer routine screening.[2, 3] The National Chlamydia Screening Programme of Public Health England recommends that “all sexually active under-25 year old men and women be tested for chlamydia annually, or on change of sexual partner (if more frequent than annually). Screening should be delivered opportunistically, i.e. sexually active young adults should be offered a test when they attend services such as GPs, community sexual and reproductive health services, pharmacies, and specialist genitourinary medicine services. Additionally, services can be provided through outreach or via self-sampling kits ordered through the internet.”[4] Guidance by Public Health England was based in part on systematic reviews on prevalence and screening effectiveness conducted in 2013 by the European Centre for Disease Prevention and Control.[5]

In 2009, the Scottish Intercollegiate Guideline Network concluded that, “In the absence of data to support a complication rate of 10% or more in women with untreated chlamydial infection, there is no evidence that a (organized) screening programme for chlamydia is cost effective with regard to reducing morbidity. Resources for testing (i.e., case finding/opportunistic testing) for chlamydia should be targeted where prevalence is known to be highest (i.e., for women, first those aged 15-19 and then those aged 20-24; for men, those under 25).”[6]

In 2011, World Health Organization HIV/AIDS programme produced guidance for the prevention and treatment of HIV and other STIs among men who have sex with men and transgender people.[7] Based on evidence for test accuracy, prevalence, and possibility of increased HIV transmission due to CT or NG infection, it recommends 1) offering periodic testing for asymptomatic urethral and rectal NG and CT infections using NAAT over not offering such testing, and 2) not offering periodic testing for asymptomatic urethral and rectal NG infections using culture (sensitivity low with 68% going undetected) over offering such testing for MSM and transgender people. Although the recommendations are intended primarily for low- and middle-income countries, the programme suggests they should be applicable to high-income countries.

**Additional File 4 References**

1. LeFevre ML. Screening for chlamydia and gonorrhea: U.S. Preventive Services Task Force recommendation statement. Ann Intern Med. 2014;161:902-10.

2. Bignell C, Fitzgerald M. UK national guideline for the management of gonorrhoea in adults, 2011. Intern J STD AIDS. 2011;22:541-47.

3. Nwokolo NC, Dragovic B, Patel S, Tong CY, Barker G, Radcliffe K. 2015 UK national guideline for the management of infection with Chlamydia trachomatis. Inter J STD AIDS. 2016;27:251-67.

4. Public Health England. Chlamydia screening: evidence summary and briefing. 2014. <https://www.gov.uk/government/publications/chlamydia-screening-evidence-summary-and-briefing> Accessed 21 June 2018.

5. European Centre for Disease Prevention and Control: Chlamydia control in Europe: literature review. Stockholm: ECDC; 2014. <https://ecdc.europa.eu/en/publications-data/chlamydia-control-europe-literature-review> Accessed 21 June 2018.

6. Scottish Intercollegiate Guidelines Network: Management of genital Chlamydia trachomatis infection: a national clinical guideline 2009. [www.sign.ac.uk/assets/sign109pdf](http://www.sign.ac.uk/assets/sign109pdf). Accessed 21 June 2018.

7. WHO HIV/AIDS Programme: Prevention and treatment of HIV and other sexually transmitted infections among men who have sex with men and transgender people. 2011. <http://www.who.int/hiv/pub/guidelines/msm_guidelines2011/en/> Accessed 21 June 2018.
