# Supplementary material for: Screening for chlamydia and/or gonorrhea in primary health care: protocol for systematic review
Source: Syst Rev. 2018 Dec 26;7:248. doi: 10.1186/s13643-018-0904-5 (PMC6307186; doi:10.1186/s13643-018-0904-5)
Supplement: Supplementary file 5 — Interpreting Evidence Comparing Universal Versus Risk-Based Screening Strategies. (DOCX 149 kb) [file 13643_2018_904_MOESM5_ESM.docx]

**Additional File 5**

**Interpreting Evidence Comparing Universal Versus Risk-Based Screening Strategies**

Screening interventions may be categorized as (i) universal, that is, testing all sexually active persons, which may be further defined by age and/or sex, or (ii) risk-based, using a strategy (e.g., “pre-screen”; algorithm or questionnaire, as defined by study authors but not only based on age or sex) in order to stratify patients into high versus not at high-risk categories. To best capture the real-world decision (and thus effectiveness) of whether or not to implement a universal versus risk-based strategy, study designs would enrol participants before assessing their level of risk, and would allocate participants (or define groups if observational study) to either the risk-based intervention group or to a control group of (ideally) universal screening (upper diagram in Figure), or no screening (upper diagram in Figure without second randomization process but rather not screening anyone in No risk-based screening strategy group). Further, authors would compare findings between study intervention groups rather than based on the participants’ risk status. The analysis would then account for there being some individuals in the risk-based strategy arm that had an infection that was not detected and/or treated (false negatives from use of risk tool), as well as providing the direct comparison with a universal screening approach.

Studies only enrolling and reporting findings for high-risk individuals (blue circles in Figure) will not be considered to adequately evaluate the utility of the risk-based strategy. Indirect comparison between studies of universal screening and studies that enrol only high-risk people will be considered, while acknowledging this does not account for the effects of screening in not-at-high-risk people. Alternatively, studies of universal screening that report on overall findings as well as those from performing interaction tests based on risk status (ideally through randomization stratified by risk to balance groups at baseline) may also be informative (lower diagram in Figure).

**Risk-based study designs offering the most direct evidence (adapted from Sargent et al. 2005^1^)**


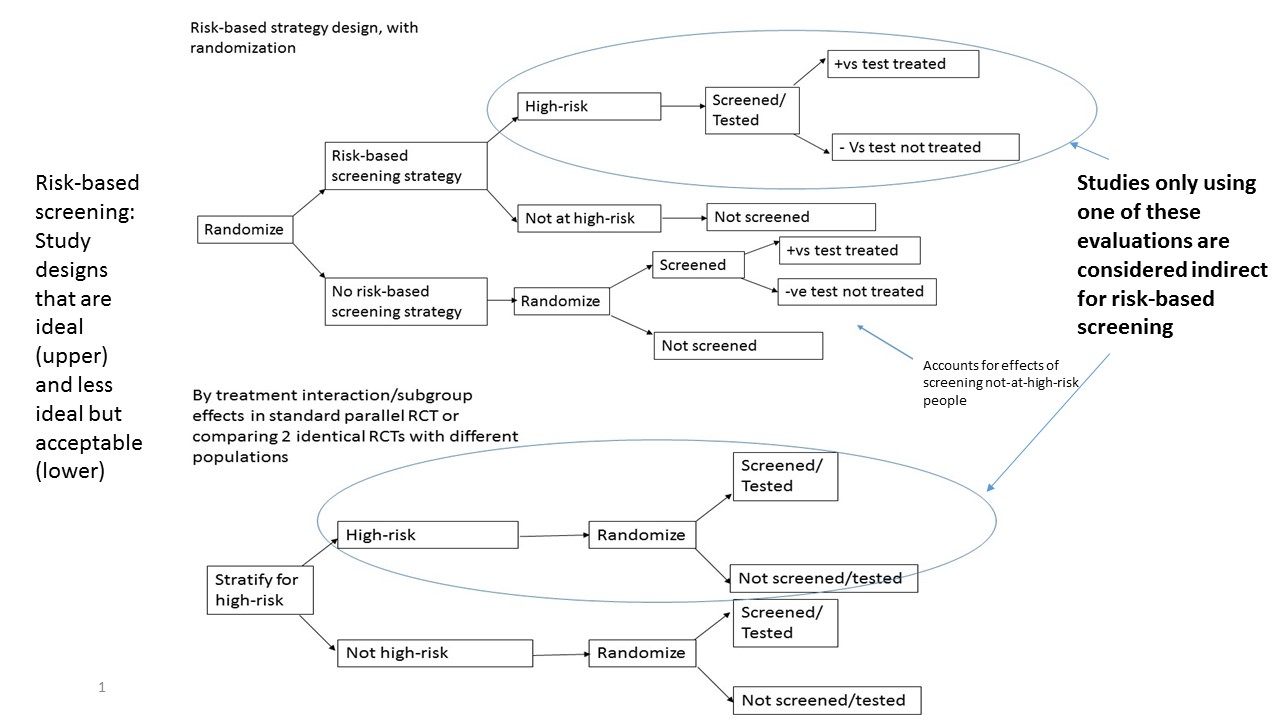


^1^ Sargent DJ, Conley BA, Allegra C, et al. Clinical trial designs for predictive marker validation in cancer treatment trials. J Clin Oncol. 2005 Mar 20;23(9):2020-7. doi: 10.1200/jco.2005.01.112. PMID: 15774793.
