# Supplementary material for: Screening for chlamydia and/or gonorrhea in primary health care: protocol for systematic review
Source: Syst Rev. 2018 Dec 26;7:248. doi: 10.1186/s13643-018-0904-5 (PMC6307186; doi:10.1186/s13643-018-0904-5)
Supplement: Supplementary file 6 — Methods for Integrating Existing Systematic Reviews into New Reviews. (DOCX 25 kb) [file 13643_2018_904_MOESM6_ESM.docx]

**Additional File 6**

**Methods for Integrating Existing Systematic Reviews into New Reviews**

One or more systematic reviews may exist that align with one or more key questions (KQs) of the reviews undertaken to inform CTFPHC guidelines. The CTFPHC and ERSCs have considered the manner in which new reviews conducted for CTFPHC guidelines can benefit from efficiencies by incorporating existing systematic reviews, while maintaining methodological rigor in their own systematic review conduct, closely aligning existing reviews within their review scope (i.e., inclusion/exclusion criteria), and maintaining consistency with other CTFPHC Methods. They have based their approach on work conducted by a methods working group composed of investigators from the Evidence-based Practice Center Program funded by the U.S. Agency for Healthcare Research and Quality.[1,2] A summary of the way the ERSCs will operationalize the 12 AHRQ recommendations (Box 1) to meet their needs is outlined below. This approach differs from situations when “updating” a single existing systematic review is deemed suitable, that is, in some cases a high-quality review will be used to answer one or more of the CTFPHC KQs in entirety, usually without revisions to the review’s scope, search for evidence (apart from updating to present), methodological quality/risk of bias assessments, data extraction, or data analysis.

**Summary of CTFPHC Approach**

The recommendations developed by AHRQ (Box 1) will serve as an overall framework for ERSC reviews, although in most cases existing systematic reviews will be used to build efficiencies in discrete steps within the review process―mainly search and selection of literature, and data extraction―which will not generally include refinement of the scope or data analysis and interpretation. Moreover, we will not in most circumstances include a systematic review itself as a study design for inclusion (unless the intention is to specifically conduct an overview of reviews). The ability to use any given systematic review will largely depend on how it aligns with the CTFPHC review’s scope (PICOTS). A further primary consideration will be the comprehensiveness of its search strategy and reporting of literature flow. It is important to note that some CTFPHC reviews need to be complex with multiple stages (e.g., a review of screening effectiveness for patient-important benefits and harms may require including evidence on indirect evidence of test accuracy and treatment) such that existing systematic reviews may exist for one or more discrete stages but not for others. Some key points on the operationalization, and minor revision, by the ERSCs of these recommendations are provided below.

1. **Choosing systematic reviews**: Following the identification of relevant reviews (a search for systematic reviews may be undertaken for some topics), the evidence for each will be mapped to the PICOTS elements and the quality of the review will be assessed (e.g., using the AMSTAR tool which has been evaluated and found effective to discriminate reviews with high and low quality of methods and reporting).^3^ Some of the CTFPHC KQs may only have a single existing systematic review for possible incorporation, while others may have more than one; if suitable, a decision between systematic reviews will be based on methodological quality, comprehensiveness and quality of its literature search and reporting (e.g., assessed using PRESS checklist), comprehensiveness of reporting on included studies, and the best fit within the CTFPHC scope and methods. In some cases two or more reviews may be integrated because, together, they capture the full scope of the CTFPHC KQ(s). Rationale will be provided for choices made.

**Note:** If no review is deemed a good fit for purpose for integration (i.e., de novo process all together appears to be best option) we will at minimum examine available reviews for their search strategies (to ensure that our search strategies are comprehensive) and review their reference lists for identification of studies.

1. **Searching**: Various strategies will be considered. If one or more reviews are fit for purpose (but do not meet criteria for classification as a systematic review update) and cover a scope that is *very similar or broader* than the CTFPHC topic, we may update the search(es) if the last search date was prior to 6 months before commencing our review. When there are multiple reviews being considered, updating the literature to present may involve a new comprehensive search strategy to identify studies published after the date of the earliest existing review; this may reduce complexities when trying to implement, document, and remove duplicates from multiple searches. Alternatively, if the scope of the existing review(s) is *narrower* (e.g., missing an element in PICOTS) or the search *deemed sub-optimal in some manner* (e.g., missing key terms, additional database viewed as highly relevant) we may re-run the existing review’s search concurrent with an original (e.g., broader) search and remove the citations previously screened for the other review. If more appropriate, we may update the other review’s search and use a new search for the missing PICO element(s) (e.g., one additional intervention) for a longer time period to meet our timeframe. In cases where we feel screening excluded studies lists is appropriate we will also undertake this. Careful consideration will be used to ensure a comprehensive search is conducted regardless of approach taken; moreover, the ERSC librarians will help determine on a case-by-case basis what approach would be feasible for implementation to ensure aims of building efficiencies are possible.
2. **Screening and selection**: We will assess articles included in all relevant reviews (based on full text if necessary) to determine if they meet our inclusion criteria.
3. **Data extraction and methodological quality assessments**: We will consider incorporating the data on study and participant characteristics rather than extracting these data anew; we may also use the review author’s risk of bias assessments if the tools/methods are consistent with CTFPHC methods. These steps will create efficiencies but because they are dependent on the quality of the systematic review and extent of reporting, the ERSC staff will verify the data on at least 5 to 10% of studies.^1^
4. **Data analysis**: We will consider using quantitative outcome data from reviews (with verification), but will not typically use meta-analyses or quality (GRADE) assessments of existing reviews.
5. **Reporting**: Transparent reporting of all integration steps used will be included in the evidence review report.

**Box 1. AHRQ recommendations on integrating existing systematic reviews for new systematic reviews.**

1. Robinson KA, Chou R, Berkman ND, et al. Integrating bodies of evidence: existing systematic reviews and primary studies. Methods Guide for Effectiveness and Comparative Effectiveness Reviews [Internet]. Rockville (MD): Agency for Healthcare Research and Quality (US); 2015 Feb.

1. Existing reviews should be confirmed as systematic reviews through the application of a minimum set of eligibility criteria. We propose that the minimum eligibility criteria for systematic reviews include an explicit and adequate search, application of predefined eligibility criteria to select studies, risk of bias assessment for included studies, and synthesis of results.

2. Criteria to assess the relevance, in terms of question elements and currency, and quality of existing systematic reviews under consideration for inclusion in reviews should be predefined.

3. The quality of relevant existing systematic reviews should be assessed in an explicit manner with a minimum set of quality criteria that include search of multiple sources, use of a generally accepted tool for risk of bias assessment, and sufficient information to assess the strength of the body of evidence that includes the major domains of risk of bias, directness, consistency, precision, and reporting bias.

4. The risk of bias assessments from the existing systematic review may be used when the review described an explicit process, including the use of a tool or method that is compatible with the approach of the current review and that assessed the key sources of potential bias.

5. We suggest that risk of bias assessment be repeated in a sample of studies from an existing review under consideration for inclusion in a new review to confirm concordance with current review team approach.

6. We recommend that at a minimum, reviews should narratively describe findings of the prior review(s), including the number and types of studies included, and the overall findings.

7. We recommend that newly identified studies be clearly distinguished from studies in the existing review(s) when presented in the narrative and any tables (eg, separate tables).

8. Summary tables should include sufficient information to support ratings for overall strength of evidence, including ratings for individual strength of evidence domains (study limitations, consistency, precision, directness, reporting bias). The strength of evidence ratings should be based on the underlying primary evidence, not the number or quality of existing systematic reviews.

9. Using strength of evidence domains as a framework (study limitations, consistency, precision, directness, and reporting bias), review authors should consider how new evidence would change estimates of effect or ratings for strength of evidence. A new quantitative synthesis (ie, pooled estimate) is needed if new studies would change conclusions or strength of evidence judgements, or to obtain a more precise or more up-to-date estimate.

10. In cases where the existing systematic review(s) did not complete strength of evidence grading for a comparison and outcome of interest, the strength of evidence should be assessed for the body of evidence, considering primary studies from prior review(s) and any new studies identified.

11. In cases where no new studies are added to the body of evidence, the strength of evidence assessment from the existing systematic review may be used if conducted using an acceptable grading approach consistent with current review context. In these cases, we suggest that the overall strength of evidence assessment be reviewed, considering the strength of evidence domains, to confirm consistency with current review team assessments.

12. In cases where new studies are added to the body of evidence, the strength of evidence may need to be reassessed on the basis of all studies/evidence.

2. Robinson KA, Chou R, Berkman ND, et al. Twelve recommendations for integrating existing systematic reviews into new reviews: EPC guidance. J Clin Epidemiol. 2016 Feb;70:38-44. PMID: 26261004.
