# Supplementary material for: Screening for chlamydia and/or gonorrhea in primary health care: protocol for systematic review
Source: Syst Rev. 2018 Dec 26;7:248. doi: 10.1186/s13643-018-0904-5 (PMC6307186; doi:10.1186/s13643-018-0904-5)
Supplement: Supplementary file 7 — Search Strategies. (DOCX 57 kb) [file 13643_2018_904_MOESM7_ESM.docx]

**Additional File 7**

**Search Strategies**

**Key Questions 1, 2, and 3 (see additional below for update from another search for KQ 3 health state utility studies)**

Database: Ovid MEDLINE(R) Epub Ahead of Print, In-Process & Other Non-Indexed Citations, Ovid MEDLINE(R) Daily and Ovid MEDLINE(R) 1946 to Present

| 1 exp *Chlamydia Infections/di (3530)  2 *Chlamydia trachomatis/ip (2437)  3 exp *Gonorrhea/di (1733)  4 *Sexually Transmitted Diseases, Bacterial/di (143)  5 or/1-4 [Coordinated concepts for chalmydia/gonorrhea/sti screening] (6375)  6 Chlamydia/ (2771)  7 exp Chlamydia Infections/ (19957)  8 Chlamydia trachomatis/ (11374)  9 Chlamydiaceae Infections/ (130)  10 Chlamydiaceae/ (158)  11 exp Gonorrhea/ (13719)  12 Neisseria gonorrhoeae/ (9251)  13 Sexually Transmitted Diseases, Bacterial/ (974)  14 chlam#dia*.tw,kf. not pneumon*.ti. (22829)  15 (gonorrh* or gonococ*).tw,kf. (22013)  16 (sexually transmitted adj1 (disease* or infection*)).ti. (7251)  17 (STD or STDs or STI or STIs).ti. (3552)  18 venereal disease*.ti. (2748)  19 or/6-18 [Combined MeSH & text words for chlamydia/gonorrhea/STIs] (59521)  20 Diagnosis/ (17067)  21 Early Diagnosis/ (22616)  22 exp Mass Screening/ (116478)  23 Self-Examination/ (1016)  24 case finding.tw,kf. (4373)  25 detect*.ti. (325877)  26 detect*.ab. /freq=2 (617698)  27 diagnose*.tw,kf. (633564)  28 diagnosis.ti. (354975)  29 diagnosis.ab. /freq=2 (332916)  30 screen*.tw,kf. (643257)  31 (self test* or selftest*).tw,kf. (1232)  32 (tested or testing).tw,kf. (1253948)  33 or/20-32 [Combined MeSH & text words for screening] (3456418)  34 and/19,33 (17884)  35 or/5,34 [Chlamydia/gonorrhea/STIs screening] (19946)  36 randomized controlled trial.pt. (462719)  37 controlled clinical trial.pt. (92450)  38 randomized.ab. (414055)  39 placebo.ab. (189720)  40 clinical trials as topic.sh. (183902)  41 randomly.ab. (291792)  42 trial.ab. (430870)  43 or/36-42 (1281877)  44 exp animals/ not humans.sh. (4465815)  45 43 not 44 [Cochrane Highly Sensitive Search Strategy for identifying randomized trials in MEDLINE: sensitivity- and precision-maximizing version (2008 revision) ; Lefebvre C, et al. Retrieved: http://handbook.cochrane.org/chapter_6/6_searching_for_studies.htm] (1174362)  46 and/35,45 [RCT filter applied to chlamydia/gonorrhea/STI screening] (1116)  47 clinical study.pt. (2889)  48 clinical trial.pt. (510675)  49 comparative study.pt. (1800136)  50 controlled clinical trial.pt. (92450)  51 evaluation studies.pt. (235164)  52 observational study.pt. (48427)  53 Choice Behavior/ (29269)  54 Clinical Studies as Topic/ (254)  55 exp Clinical Trials as Topic/ (314840)  56 exp Cohort Studies/ (1749726)  57 Decision Making/ (83815)  58 exp Evaluation Studies as Topic/ (951998)  59 Focus Groups/ (24697)  60 exp Health Care Surveys/ (31768)  61 Health Surveys/ (57145)  62 Interviews as Topic/ (54494)  63 Narration/ (7137)  64 Observational Studies as Topic/ (3073)  65 Patient Acceptance of Health Care/ (39652)  66 Patient Preference/ (6315)  67 Qualitative Research/ (39485)  68 "Surveys and Questionnaires"/ (400154)  69 Self Report/ (23397)  70 ((attitude* or choice? or choos* or chose? or decid* or decis* or preference*) adj3 (client* or female* or male* or men or patient* or wom#n* or trans-gender* or transgender*)).ti,ab. (78217)  71 case series*.ti,ab. (59661)  72 clinical trial.ti,ab. (116777)  73 cohort*.ti,ab. (463057)  74 comparative stud*.ti,ab. (93205)  75 control.ti,ab. (2264888)  76 controlled.ti,ab. (665752)  77 descriptive stud*.ti,ab. (23228)  78 ((design* or trial*) adj3 (step* adj wedge*)).ti,ab. (449)  79 ((discussion* or interview* or questionnaire*) adj3 ("face to face" or guide or "in depth" or indepth or informal or semistructured or structured or unstructured)).ti,ab. (101481)  80 ethnograph*.ti,ab. (9059)  81 (field work or fieldwork).ti,ab. (3985)  82 focus group*.ti,ab. (36857)  83 ((follow up or followup) adj stud*).ti,ab. (45973)  84 key informant*.ti,ab. (5926)  85 longitudinal.ti,ab. (205803)  86 multidimensional stud*.ti,ab. (120)  87 (non-random* or nonrandom*).ti,ab. (33626)  88 prospective*.ti,ab. (604485)  89 qualitative.ti,ab. (184473)  90 quasirandom*.ti,ab. (138)  91 questionnaire*.ti,ab. (431983)  92 retrospective*.ti,ab. (602029)  93 volunteer*.ti,ab. (177027)  94 or/47-93 (7882782)  95 exp Animals/ not Humans/ (4465815)  96 94 not 95 [Non-Randomized & Qualitative Studies filter - not validated] (6748703)  97 and/35,96 [Non-Randomized & Qualitative Studies filter applied to chlamydia/gonorrhea/STI screening] (9590)  98 or/46,97 [RCT & Non-Randomized & Qualitative chlamydia/gonorrhea/STI screening studies] (9733)  99 (1996* or 1997* or 1998* or 1999* or 2*).dt. (17427247)  100 98 and 99 [Date limit applied] (7184)  101 limit 100 to (english or french) (6795) |
| --- |

Database: Ovid Embase 1996 to 2018 Week 22

| 1 Chlamydia/ (6133)  2 Chlamydia trachomatis/ (11577)  3 chlamydiaceae/ (462)  4 exp Chlamydiaceae infection/ (16671)  5 exp chlamydiasis/ (15150)  6 exp gonorrhea/ (9867)  7 neisseria gonorrhoeae/ (8061)  8 chlam#dia*.tw,kw. not pneumon*.ti. (20152)  9 (gonorrh* or gonococ*).tw,kw. (12497)  10 (sexually transmitted adj1 (disease* or infection*)).ti. (6521)  11 (STD or STDs or STI or STIs).ti. (4327)  12 venereal disease*.ti. (184)  13 or/1-12 [Combined Emtree & text words for chlamydia/gonorrhea/STIs] (44120)  14 mass screening/ (35350)  15 self examination/ (2366)  16 screening/ (162065)  17 case finding.tw,kw. (4172)  18 detect*.ti. (281832)  19 detect*.ab. /freq=2 (677811)  20 diagnose*.tw,kw. (870702)  21 diagnosis.ti. (239734)  22 diagnosis.ab. /freq=2 (471309)  23 screen*.tw,kw. (781162)  24 (self test* or selftest*).tw,kw. (1552)  25 (tested or testing).tw,kw. (1345345)  26 or/14-25 [Combined Emtree & text words for screening] (3708962)  27 and/13,26 (17733) [Chlamydia/gonorrhea/STIs screening]  28 crossover-procedure/ (51132)  29 double-blind procedure/ (123335)  30 randomized controlled trial/ (455398)  31 single-blind procedure/ (30016)  32 allocat*.tw. (113267)  33 assign*.tw. (293044)  34 (cross over* or crossover*).tw. (73059)  35 (doubl* adj blind*).tw. (141929)  36 factorial*.tw. (28731)  37 placebo*.tw. (224018)  38 random*.tw. (1167849)  39 (single* adj blind*).tw. (17670)  40 volunteer*.tw. (179844)  41 or/28-40 (1725262)  42 exp animal/ not human/ (2655382)  43 41 not 42 [Recommended Emtree & Embase text words for identifying trials in Cochrane Handbook (2008 revision) Chapter 6.3.2.2] (1557605)  44 and/27,43 [RCT filter applied to chlamydia/gonorrhea/STI screening] (1484)  45 clinical study/ (101802)  46 exp clinical trial/ (1166463)  47 exp "clinical trial (topic)"/ (268100)  48 cohort analysis/ (368295)  49 comparative study/ (546650)  50 controlled clinical trial/ (415572)  51 decision making/ (169226)  52 evaluation study/ (32996)  53 health care survey/ (13555)  54 health survey/ (164814)  55 interview/ (157490)  56 narrative/ (7174)  57 observational study/ (140063)  58 patient attitude/ (53374)  59 patient preference/ (13822)  60 qualitative research/ (53218)  61 questionnaire/ (539570)  62 self report/ (97850)  63 ((attitude* or choice? or choos* or chose? or decid* or decis* or preference*) adj3 (client* or female* or male* or men or patient* or wom#n* or trans-gender* or transgender*)).ti,ab. (99713)  64 case series*.ti,ab. (79528)  65 clinical trial.ti,ab. (147889)  66 cohort*.ti,ab. (740990)  67 comparative stud*.ti,ab. (72816)  68 control.ti,ab. (2375053)  69 controlled.ti,ab. (726770)  70 descriptive stud*.ti,ab. (31601)  71 ((design* or trial*) adj3 (step* adj wedge*)).ti,ab. (580)  72 ((discussion* or interview* or questionnaire*) adj3 ("face to face" or guide or "in depth" or indepth or informal or semistructured or structured or unstructured)).ti,ab. (121867)  73 ethnograph*.ti,ab. (8927)  74 (field work or fieldwork).ti,ab. (3769)  75 focus group*.ti,ab. (44083)  76 ((follow up or followup) adj stud*).ti,ab. (43437)  77 key informant*.ti,ab. (6510)  78 longitudinal.ti,ab. (236540)  79 multidimensional stud*.ti,ab. (115)  80 (non-random* or nonrandom*).ti,ab. (37100)  81 prospective*.ti,ab. (822132)  82 qualitative.ti,ab. (196175)  83 quasirandom*.ti,ab. (155)  84 questionnaire*.ti,ab. (568077)  85 retrospective*.ti,ab. (910552)  86 volunteer*.ti,ab. (179844)  87 or/45-86 (7155683)  88 exp animal/ not human/ (2655382)  89 87 not 88 [Non-Randomized & Qualitative Studies filter - not validated] (6439665)  90 and/27,89 [Non-Randomized & Qualitative Studies filter applied to chlamydia/gonorrhea/STI screening] (8174)  91 or/44,90 [RCT & Non-Randomized & Qualitative chlamydia/gonorrhea/STI screening studies] (8455)  92 limit 91 to (english or french) (8070) |
| --- |

Database: Wiley Cochrane Library

| #1 [mh ^Chlamydia] 35  #2 [mh "Chlamydia Infections"] 684  #3 [mh ^"Chlamydia trachomatis"] 379  #4 [mh ^"Chlamydiaceae Infections"] 1  #5 [mh ^Chlamydiaceae] 0  #6 [mh Gonorrhea] 455  #7 [mh ^"Neisseria gonorrhoeae"] 182  #8 [mh ^"Sexually Transmitted Diseases, Bacterial"] 25  #9 chlam?dia*:ti,ab,kw not pneumon*:ti 1374  #10 (gonorrh* or gonococ*):ti,ab,kw 1344  #11 (sexually transmitted near/1 (disease* or infection*)):ti,ab,kw 1780  #12 (STD or STDs or STI or STIs):ti 308  #13 "venereal disease*":ti 14  #14 {or #1-#13} 3695  #15 [mh ^Diagnosis] 153  #16 [mh ^"Early Diagnosis"] 618  #17 [mh "Mass Screening"] 5815  #18 [mh ^Self-Examination] 116  #19 "case finding":ti,ab,kw 284  #20 detect*:ti 7281  #21 diagnose*:ti,ab,kw 34563  #22 diagnosis:ti 6128  #23 screen*:ti,ab,kw 37724  #24 ("self test*" or selftest*):ti,ab,kw 167  #25 (tested or testing):ti,ab,kw 73551  #26 {or #15-#25} 144252  #27 #14 and #26 1182  #28 #14 and #26 Publication Year from 1996 to 2018 1034 |
| --- |

Database: CINAHL Plus with Full Text via EBSCOhost

| \| **#** \| **Query** \| **Limiters/Expanders** \| **Results** \| \| --- \| --- \| --- \| --- \| \| S1 \| (MH "Chlamydia+") \| Search modes - Find all my search terms \| 1,701 \| \| S2 \| (MH "Chlamydia Infections+") \| Search modes - Find all my search terms \| 3,847 \| \| S3 \| (MH "Chlamydiaceae") \| Search modes - Find all my search terms \| 4 \| \| S4 \| (MH "Chlamydiaceae Infections") \| Search modes - Find all my search terms \| 3 \| \| S5 \| (MH "Gonorrhea") \| Search modes - Find all my search terms \| 2,369 \| \| S6 \| (MH "Neisseria Infections") \| Search modes - Find all my search terms \| 132 \| \| S7 \| (MH "Sexually Transmitted Diseases, Bacterial") \| Search modes - Find all my search terms \| 232 \| \| S8 \| (TI chlam?dia* or AB chlam?dia*) NOT TI pneumon* \| Search modes - Find all my search terms \| 3,944 \| \| S9 \| TI (gonorrh* or gonococ*) or AB (gonorrh* or gonococ*) \| Search modes - Find all my search terms \| 2,897 \| \| S10 \| TI ("sexually transmitted" N1 (disease* or infection*)) \| Search modes - Find all my search terms \| 3,064 \| \| S11 \| TI (STD or STDs or STI or STIs) \| Search modes - Find all my search terms \| 2,365 \| \| S12 \| TI "venereal disease*" \| Search modes - Find all my search terms \| 135 \| \| S13 \| S1 OR S2 OR S3 OR S4 OR S5 OR S6 OR S7 OR S8 OR S9 OR S10 OR S11 OR S12 \| Search modes - Find all my search terms \| 11,335 \| \| S14 \| (MH "Diagnosis") \| Search modes - Find all my search terms \| 7,290 \| \| S15 \| (MH "Early Diagnosis") \| Search modes - Find all my search terms \| 6,135 \| \| S16 \| (MH "Health Screening") \| Search modes - Find all my search terms \| 35,310 \| \| S17 \| (MH "Home Diagnostic Tests") \| Search modes - Find all my search terms \| 573 \| \| S18 \| (MH "Self Assessment") \| Search modes - Find all my search terms \| 7,618 \| \| S19 \| TI ("case finding") or AB ("case finding") \| Search modes - Find all my search terms \| 1,036 \| \| S20 \| TI diagnose* or AB diagnose* \| Search modes - Find all my search terms \| 125,827 \| \| S21 \| TI diagnosis \| Search modes - Find all my search terms \| 49,546 \| \| S22 \| TI screen* or AB screen* \| Search modes - Find all my search terms \| 121,705 \| \| S23 \| TI ("self test*" or selftest*) or AB ("self test*" or selftest*) \| Search modes - Find all my search terms \| 720 \| \| S24 \| TI (tested or testing) or AB (tested or testing) \| Search modes - Find all my search terms \| 171,259 \| \| S25 \| S14 OR S15 OR S16 OR S17 OR S18 OR S19 OR S20 OR S21 OR S22 OR S23 OR S24 \| Search modes - Find all my search terms \| 451,693 \| \| S26 \| S13 AND S25 \| Search modes - Find all my search terms \| 3,907 \| \| S27 \| (MH "Random Assignment") \| Search modes - Find all my search terms \| 48,233 \| \| S28 \| PT Clinical trial \| Search modes - Find all my search terms \| 85,846 \| \| S29 \| PT Randomized Controlled Trial \| Search modes - Find all my search terms \| 82,172 \| \| S30 \| TX allocat* random* \| Search modes - Find all my search terms \| 31,534 \| \| S31 \| TX clinic* n1 trial* \| Search modes - Find all my search terms \| 280,372 \| \| S32 \| TX ( (doubl* n1 blind*) or (doubl* n1 mask*) ) \| Search modes - Find all my search terms \| 964,144 \| \| S33 \| TX placebo* \| Search modes - Find all my search terms \| 83,131 \| \| S34 \| TX random* allocat* \| Search modes - Find all my search terms \| 31,534 \| \| S35 \| TX randomi* control* trial* \| Search modes - Find all my search terms \| 244,802 \| \| S36 \| TX ( (singl* n1 blind*) or (singl* n1 mask*) ) \| Search modes - Find all my search terms \| 16,967 \| \| S37 \| TX ( (trebl* n1 blind*) or (trebl* n1 mask*) ) \| Search modes - Find all my search terms \| 9 \| \| S38 \| TX ( (tripl* n1 blind*) or (tripl* n1 mask*) ) \| Search modes - Find all my search terms \| 483 \| \| S39 \| S27 OR S28 OR S29 OR S30 OR S31 OR S32 OR S33 OR S34 OR S35 OR S36 OR S37 OR S38 \| Search modes - Find all my search terms \| 1,302,135 \| \| S40 \| ((MH "Vertebrates+") NOT MH Human) \| Search modes - Find all my search terms \| 148,882 \| \| S41 \| S39 NOT S40 \| Search modes - Find all my search terms \| 1,280,954 \| \| S42 \| S26 AND S41 \| Search modes - Find all my search terms \| 777 \| \| S43 \| (MH "Decision Making") \| Search modes - Find all my search terms \| 38,157 \| \| S44 \| (MH "Decision Making, Patient") \| Search modes - Find all my search terms \| 12,832 \| \| S45 \| (MH "Interviews+") \| Search modes - Find all my search terms \| 182,715 \| \| S46 \| (MH "Narratives+") \| Search modes - Find all my search terms \| 14,914 \| \| S47 \| (MH "Nonexperimental Studies") \| Search modes - Find all my search terms \| 18,388 \| \| S48 \| (MH "Prospective Studies+") \| Search modes - Find all my search terms \| 348,597 \| \| S49 \| (MH "Questionnaires+") \| Search modes - Find all my search terms \| 331,580 \| \| S50 \| (MH "Self Report+") \| Search modes - Find all my search terms \| 57,213 \| \| S51 \| (MH "Surveys") \| Search modes - Find all my search terms \| 117,727 \| \| S52 \| TI ((attitude* or choice* or choos* or chose* or decid* or decis* or preference*) N3 (client* or female* or male* or men or patient* or wom?n* or "trans-gender*" or transgender*)) or AB ((attitude* or choice* or choos* or chose* or decid* or decis* or preference*) N3 (client* or female* or male* or men or patient* or wom?n* or "trans-gender*" or transgender*)) \| Search modes - Find all my search terms \| 34,767 \| \| S53 \| TI "clinical trial" or AB "clinical trial" \| Search modes - Find all my search terms \| 33,087 \| \| S54 \| TI cohort* or AB cohort* \| Search modes - Find all my search terms \| 143,449 \| \| S55 \| TI "comparative stud*" or AB "comparative stud*" \| Search modes - Find all my search terms \| 10,655 \| \| S56 \| TI control or AB control \| Search modes - Find all my search terms \| 344,154 \| \| S57 \| TI controlled or AB controlled \| Search modes - Find all my search terms \| 144,743 \| \| S58 \| TI "descriptive stud*" or AB "descriptive stud*" \| Search modes - Find all my search terms \| 13,449 \| \| S59 \| TI ((design* or trial*) N3 (step* N1 wedge*)) or AB ((design* or trial*) N3 (step* N1 wedge*)) \| Search modes - Find all my search terms \| 300 \| \| S60 \| TI ((discussion* or interview* or questionnaire*) N3 ("face to face" or guide or "in depth" or indepth or informal or semistructured or structured or unstructured)) or AB ((discussion* or interview* or questionnaire*) N3 ("face to face" or guide or "in depth" or indepth or informal or semistructured or structured or unstructured)) \| Search modes - Find all my search terms \| 58,734 \| \| S61 \| TI ethnograph* or AB ethnograph* \| Search modes - Find all my search terms \| 7,110 \| \| S62 \| TI ("field work" or fieldwork) or AB ("field work" or fieldwork) \| Search modes - Find all my search terms \| 2,306 \| \| S63 \| TI "focus group*" or AB "focus group*" \| Search modes - Find all my search terms \| 24,884 \| \| S64 \| TI ("key informant*") or AB ("key informant*") \| Search modes - Find all my search terms \| 3,149 \| \| S65 \| TI longitudinal or AB longitudinal \| Search modes - Find all my search terms \| 61,313 \| \| S66 \| TI ("multidimensional stud*") or AB ("multidimensional stud*") \| Search modes - Find all my search terms \| 36 \| \| S67 \| TI ("non-random*" or nonrandom*) or AB ("non-random*" or nonrandom*) \| Search modes - Find all my search terms \| 6,757 \| \| S68 \| TI prospective* or AB prospective* \| Search modes - Find all my search terms \| 151,752 \| \| S69 \| TI qualitative or AB qualitative \| Search modes - Find all my search terms \| 87,233 \| \| S70 \| TI quasirandom* or AB quasirandom* \| Search modes - Find all my search terms \| 45 \| \| S71 \| TI questionnaire* or AB questionnaire* \| Search modes - Find all my search terms \| 155,466 \| \| S72 \| TI retrospective* or AB retrospective* \| Search modes - Find all my search terms \| 134,149 \| \| S73 \| TI volunteer* or AB volunteer* \| Search modes - Find all my search terms \| 32,900 \| \| S74 \| S43 OR S44 OR S45 OR S46 OR S47 OR S48 OR S49 OR S50 OR S51 OR S52 OR S53 OR S54 OR S55 OR S56 OR S57 OR S58 OR S59 OR S60 OR S61 OR S62 OR S63 OR S64 OR S65 OR S66 OR S67 OR S68 OR S69 OR S70 OR S71 OR S72 OR S73 \| Search modes - Find all my search terms \| 1,487,320 \| \| S75 \| ((MH "Vertebrates+") NOT MH Human) \| Search modes - Find all my search terms \| 148,882 \| \| S76 \| S74 NOT S75 \| Search modes - Find all my search terms \| 1,454,546 \| \| S77 \| S26 and S76 \| Search modes - Find all my search terms \| 1,864 \| \| S78 \| S42 OR S77 \| Search modes - Find all my search terms \| 2,185 \| \| S79 \| S42 OR S77 \| Limiters - Published Date: 19960101-20181231; Language: English, French  Search modes - Find all my search terms \| 2,129 \| |
| --- | --- | --- | --- | --- | --- | --- | --- | --- | --- | --- | --- | --- | --- | --- | --- | --- | --- | --- | --- | --- | --- | --- | --- | --- | --- | --- | --- | --- | --- | --- | --- | --- | --- | --- | --- | --- | --- | --- | --- | --- | --- | --- | --- | --- | --- | --- | --- | --- | --- | --- | --- | --- | --- | --- | --- | --- | --- | --- | --- | --- | --- | --- | --- | --- | --- | --- | --- | --- | --- | --- | --- | --- | --- | --- | --- | --- | --- | --- | --- | --- | --- | --- | --- | --- | --- | --- | --- | --- | --- | --- | --- | --- | --- | --- | --- | --- | --- | --- | --- | --- | --- | --- | --- | --- | --- | --- | --- | --- | --- | --- | --- | --- | --- | --- | --- | --- | --- | --- | --- | --- | --- | --- | --- | --- | --- | --- | --- | --- | --- | --- | --- | --- | --- | --- | --- | --- | --- | --- | --- | --- | --- | --- | --- | --- | --- | --- | --- | --- | --- | --- | --- | --- | --- | --- | --- | --- | --- | --- | --- | --- | --- | --- | --- | --- | --- | --- | --- | --- | --- | --- | --- | --- | --- | --- | --- | --- | --- | --- | --- | --- | --- | --- | --- | --- | --- | --- | --- | --- | --- | --- | --- | --- | --- | --- | --- | --- | --- | --- | --- | --- | --- | --- | --- | --- | --- | --- | --- | --- | --- | --- | --- | --- | --- | --- | --- | --- | --- | --- | --- | --- | --- | --- | --- | --- | --- | --- | --- | --- | --- | --- | --- | --- | --- | --- | --- | --- | --- | --- | --- | --- | --- | --- | --- | --- | --- | --- | --- | --- | --- | --- | --- | --- | --- | --- | --- | --- | --- | --- | --- | --- | --- | --- | --- | --- | --- | --- | --- | --- | --- | --- | --- | --- | --- | --- | --- | --- | --- | --- | --- | --- | --- | --- | --- | --- | --- | --- | --- | --- | --- | --- | --- | --- | --- | --- | --- | --- | --- | --- | --- | --- | --- | --- | --- | --- | --- | --- | --- | --- | --- | --- | --- | --- | --- | --- | --- | --- | --- | --- | --- | --- |

Database: Ovid PsycINFO 1987 to May Week 3 2018

| 1 gonorrhea/ (137)  2 sexually transmitted diseases/ (4128)  3 chlam#dia*.ti,ab. not pneumon*.ti. (852)  4 (gonorrh* or gonococ*).ti,ab. (684)  5 (sexually transmitted adj1 (disease* or infection*)).ti. (1258)  6 (STD or STDs or STI or STIs).ti. (1032)  7 venereal disease*.ti. (9)  8 or/1-7 [Combined subject headings & text words for chlamydia/gonorrhea/STIs] (4792)  9 health screening/ (2905)  10 medical diagnosis/ (6649)  11 screening/ (8687)  12 screening tests/ (4952)  13 "self-examination (medical)"/ (451)  14 case finding.ti,ab. (560)  15 detect*.ti. (14697)  16 detect*.ab. /freq=2 (28296)  17 diagnose*.ti,ab. (93003)  18 diagnosis.ti. (15319)  19 diagnosis.ab. /freq=2 (32636)  20 screen*.ti,ab. (78919)  21 (self test* or selftest*).ti,ab. (385)  22 (tested or testing).ti,ab. (242039)  23 or/9-22 [Combined subject headings & text words for screening] (448358)  24 and/8,23 [Chlamydia/gonorrhea/STIs screening] (1709)  25 clinical trials/ (10858)  26 (random* or sham or placebo*).ti,ab. (183742)  27 ((singl* or doubl*) adj (blind* or dumm* or mask*)).ti,ab. (21237)  28 ((tripl* or trebl*) adj (blind* or dumm* or mask*)).ti,ab. (55)  29 or/25-28 [RCT filter modified from: Strings attached: CADTH database search filters. Ottawa: CADTH; 2016. Available from: /resources/finding-evidence] (189931)  30 exp animals/ not humans.sh. (253155)  31 29 not 30 (177956)  32 24 and 31 [RCT filter applied to chlamydia/gonorrhea/STI screening] (181)  33 between groups design/ (106)  34 choice behavior/ (14083)  35 client attitudes/ (14091)  36 cohort analysis/ (1242)  37 decision making/ (62123)  38 experimental design/ (8058)  39 followup studies/ (8909)  40 interviewing/ (2518)  41 interviews/ (6368)  42 exp longitudinal studies/ (12547)  43 narratives/ (17698)  44 observation methods/ (3208)  45 preference measures/ (225)  46 preferences/ (12590)  47 qualitative research/ (7746)  48 quasi experimental methods/ (115)  49 questionnaires/ (15039)  50 self-report/ (14524)  51 exp surveys/ (7417)  52 ((attitude* or choice? or choos* or chose? or decid* or decis* or preference*) adj3 (client* or female* or male* or men or patient* or wom#n* or trans-gender* or transgender*)).ti,ab. (28320)  53 case series*.ti,ab. (3433)  54 clinical trial.ti,ab. (11791)  55 cohort*.ti,ab. (64498)  56 comparative stud*.ti,ab. (9714)  57 control.ti,ab. (335484)  58 controlled.ti,ab. (97296)  59 descriptive stud*.ti,ab. (6301)  60 ((design* or trial*) adj3 (step* adj wedge*)).ti,ab. (59)  61 ((discussion* or interview* or questionnaire*) adj3 ("face to face" or guide or "in depth" or indepth or informal or semistructured or structured or unstructured)).ti,ab. (86229)  62 ethnograph*.ti,ab. (23823)  63 (field work or fieldwork).ti,ab. (6076)  64 focus group*.ti,ab. (30105)  65 ((follow up or followup) adj stud*).ti,ab. (9352)  66 key informant*.ti,ab. (2925)  67 longitudinal.ti,ab. (91510)  68 multidimensional stud*.ti,ab. (211)  69 (non-random* or nonrandom*).ti,ab. (3797)  70 prospective*.ti,ab. (58150)  71 qualitative.ti,ab. (133792)  72 quasirandom*.ti,ab. (29)  73 questionnaire*.ti,ab. (219238)  74 retrospective*.ti,ab. (36303)  75 volunteer*.ti,ab. (30543)  76 or/33-75 (1108620)  77 exp Animals/ not Humans/ (253155)  78 76 not 77 [Non-Randomized & Qualitative Studies filter - not validated] (1052697)  79 24 and 78 [Non-Randomized & Qualitative Studies filter applied to chlamydia/gonorrhea/STI screening] (825)  80 or/32,79 [RCT & Non-Randomized & Qualitative chlamydia/gonorrhea/STI screening studies] (867)  81 limit 80 to yr="1996-Current" (841)  82 limit 81 to (english or french) (832) |
| --- |

**Key Question 3: health state utility values (update of Jackson et al. Value Health. 2014:116-130**

Database: Ovid MEDLINE(R) Epub Ahead of Print, In-Process & Other Non-Indexed Citations, Ovid MEDLINE(R) Daily and Ovid MEDLINE(R) 1946 to Present

| 1 chlamydia.mp. or exp Chlamydia Infections/ or exp Chlamydia/ or exp Chlamydia trachomatis/ (32014)  2 gonorrhea.mp. or exp Gonorrhea/ (15876)  3 "pelvic inflammatory disease".mp. or exp Pelvic Inflammatory Disease/ or PID.mp. (15328)  4 cervicitis.mp. or exp Uterine Cervicitis/ (3344)  5 ectopic pregnancy.mp. or exp Pregnancy, Ectopic/ (16593)  6 epididymitis.mp. or Epididymitis/ (3232)  7 exp Pelvic Pain/ or "chronic pelvic pain".mp. (9776)  8 Infertility, Female/ or tubal infertility.mp. (27108)  9 tubal factor infertility.mp. (433)  10 life quality.mp. (5815)  11 (hql or qol or HRQL or HRQOL).mp. (46114)  12 (QALY$ or "Quality adjusted Life Year$").mp. or Quality-Adjusted Life Years/ (15833)  13 Health Status/ or health state$.mp. (77410)  14 (utilit$ or health utilit$).mp. (176853)  15 disutility.mp. (307)  16 (sf 8 or sf8 or "short form 8" or shortform 8 or sf eight or "short form eight" or "shortform eight").tw. (461)  17 (sf 12 or sf12 or "short form 12" or shortform 12 or sf twelve or "short form twelve" or "shortform twelve").tw. (4765)  18 (sf 36 or sf36 or "short form 36" or shortform 36 or sf thirtysix or "sf thirty six" or "short form thirty six" or "short form thirtysix" or "shortform thirty six" or "shortform thirtysix").tw. (22456)  19 (sf 6d or sf6d or "short form 6d" or shortform 6d or sf six or "short form six" or "shortform six").tw. (704)  20 hui$.mp. (6477)  21 (euro qol or euro qol or eq5d or eq 5d).mp. (6498)  22 standard gamble.mp. (793)  23 ("time trade off" or tto).mp. (1481)  24 (preference$ or valuation$).mp. (148525)  25 or/1-9 (110622)  26 or/10-24 (469148)  27 25 and 26 [Combined PID Outcomes & HUI concepts from Jackson SR] (1359)  28 (2013* or 2014* or 2015* or 2016* or 2017* or 2018*).dt. (6201410)  29 27 and 28 [Date limit applied from 2013-2018] (454)  30 remove duplicates from 29 (444) |
| --- |

Database: Ovid Embase 1996 to 2018 Week 22

| 1 chlamydia.mp. or exp chlamydiasis/ or Chlamydia/ or Chlamydia trachomatis/ (30090)  2 gonorrhea.mp. or exp gonorrhea/ (10771)  3 "pelvic inflammatory disease".mp. or exp pelvic inflammatory disease/ or PID.mp. (13954)  4 cervicitis.mp. or uterine cervicitis/ (3062)  5 ectopic pregnancy.mp. or exp ectopic pregnancy/ (12973)  6 epididymitis.mp. or epididymitis/ (2788)  7 exp pelvic pain/ or "chronic pelvic pain".mp. (16201)  8 exp female infertility/ or tubal infertility.mp. (31666)  9 tubal factor infertility.mp. (562)  10 life quality.mp. (11046)  11 (hql or qol or HRQL or HRQOL).mp. (81014)  12 (QALY$ or "Quality adjusted Life Year$").mp. or quality adjusted life year/ (26484)  13 health status/ or health state$.mp. (105905)  14 (utilit$ or health utilit$).mp. (221163)  15 disutility.mp. (536)  16 (sf 8 or sf8 or "short form 8" or shortform 8 or sf eight or "short form eight" or "shortform eight").tw. (734)  17 (sf 12 or sf12 or "short form 12" or shortform 12 or sf twelve or "short form twelve" or "shortform twelve").tw. (7553)  18 (sf 36 or sf36 or "short form 36" or shortform 36 or sf thirtysix or "sf thirty six" or "short form thirty six" or "short form thirtysix" or "shortform thirty six" or "shortform thirtysix").tw. (35217)  19 (sf 6d or sf6d or "short form 6d" or shortform 6d or sf six or "short form six" or "shortform six").tw. (1252)  20 hui$.mp. (7353)  21 (euro qol or euro qol or eq5d or eq 5d).mp. (12808)  22 standard gamble.mp. (973)  23 ("time trade off" or tto).mp. (2082)  24 (preference$ or valuation$).mp. (155808)  25 or/1-9 (105699)  26 or/10-24 (595749)  27 25 and 26 [Combined PID Outcomes & HUI concepts from Jackson SR] (2658)  28 limit 27 to yr="2013-Current" (1247)  29 remove duplicates from 28 (1206) |
| --- |

Database: Wiley Cochrane Library

| #1 chlamydia:ti,ab,kw or [mh "Chlamydia Infections"] or [mh Chlamydia] or [mh "Chlamydia trachomatis"] 1566  #2 gonorrhea:ti,ab,kw or [mh Gonorrhea] 917  #3 "pelvic inflammatory disease":ti,ab,kw or [mh "Pelvic Inflammatory Disease"] or PID:ti,ab,kw 1073  #4 cervicitis:ti,ab,kw or [mh "Uterine Cervicitis"] 220  #5 "ectopic pregnancy":ti,ab,kw or [mh "Pregnancy, Ectopic"] 557  #6 epididymitis:ti,ab,kw or [mh Epididymitis] 76  #7 [mh "Pelvic Pain"] or "chronic pelvic pain":ti,ab,kw 1278  #8 [mh "Infertility, Female"] or "tubal infertility":ti,ab,kw 1433  #9 "tubal factor infertility":ti,ab,kw 32  #10 "life quality":ti,ab,kw 2376  #11 (hql or qol or HRQL or HRQOL):ti,ab,kw 13659  #12 (QALY* or "Quality adjusted Life Year*"):ti,ab,kw or [mh ^"Quality-Adjusted Life Years"] 6597  #13 [mh ^"Health Status"] or health state*:ti,ab,kw 26073  #14 (utilit* or "health utilit" *):ti,ab,kw 11844  #15 disutility:ti,ab,kw 26  #16 ("sf 8" or sf8 or "short form 8" or "shortform 8" or "sf eight" or "short form eight" or "shortform eight"):ti,ab 84  #17 ("sf 12" or sf12 or "short form 12" or "shortform 12" or "sf twelve" or "short form twelve" or "shortform twelve"):ti,ab 1250  #18 ("sf 36" or sf36 or "short form 36" or "shortform 36" or "sf thirtysix" or "sf thirty six" or "short form thirty six" or "short form thirtysix" or "shortform thirty six" or "shortform thirtysix"):ti,ab 7010  #19 ("sf 6d" or sf6d or "short form 6d" or "shortform 6d" or "sf six" or "short form six" or "shortform six"):ti,ab 221  #20 hui*:ti,ab,kw 1029  #21 ("euro qol" or "euro qol" or eq5d or "eq 5d"):ti,ab,kw 2838  #22 "standard gamble":ti,ab,kw 97  #23 ("time trade off" or tto):ti,ab,kw 200  #24 (preference* or valuation*):ti,ab,kw 11640  #25 {or #1-#9} 6318  #26 {or #10-#24} 71835  #27 #25 and #26 371  #28 #25 and #26 Publication Year from 2013 to 2018 169 |
| --- |

Database: CINAHL Plus with Full Text via EBSCOhost

| \| **#** \| **Query** \| **Limiters/Expanders** \| **Results** \| \| --- \| --- \| --- \| --- \| \| S1 \| chlamydia or (MH "Chlamydia+") or (MH "Chlamydia Infections+") or (MH "Chlamydia Trachomatis") \| Search modes - Find all my search terms \| 5,423 \| \| S2 \| gonorrhea* or (MH "Gonorrhea+") \| Search modes - Find all my search terms \| 2,956 \| \| S3 \| "pelvic inflammatory disease" or (MH "Pelvic Inflammatory Disease+") or PID \| Search modes - Find all my search terms \| 1,491 \| \| S4 \| cervicitis or (MH "Uterine Cervicitis") \| Search modes - Find all my search terms \| 212 \| \| S5 \| "ectopic pregnancy" or (MH "Pregnancy, Ectopic") \| Search modes - Find all my search terms \| 2,246 \| \| S6 \| epididymitis or (MH "Epididymitis") \| Search modes - Find all my search terms \| 285 \| \| S7 \| (MH "Pelvic Pain+") or "chronic pelvic pain" \| Search modes - Find all my search terms \| 3,415 \| \| S8 \| (MH "Infertility") or "tubal infertility" \| Search modes - Find all my search terms \| 6,944 \| \| S9 \| "tubal factor infertility" \| Search modes - Find all my search terms \| 48 \| \| S10 \| "life quality" \| Search modes - Find all my search terms \| 1,457 \| \| S11 \| hql or qol or HRQL or HRQOL \| Search modes - Find all my search terms \| 16,941 \| \| S12 \| (QALY* or "Quality adjusted Life Year*") or (MH "Quality-Adjusted Life Years") \| Search modes - Find all my search terms \| 5,815 \| \| S13 \| (MH "Health Status") or health state* \| Search modes - Find all my search terms \| 271,558 \| \| S14 \| utilit* or "health utilit*" \| Search modes - Find all my search terms \| 34,138 \| \| S15 \| disutility \| Search modes - Find all my search terms \| 153 \| \| S16 \| TI ("sf 8" or sf8 or "short form 8" or "shortform 8" or "sf eight" or "short form eight" or "shortform eight") or AB ("sf 8" or sf8 or "short form 8" or "shortform 8" or "sf eight" or "short form eight" or "shortform eight") \| Search modes - Find all my search terms \| 31 \| \| S17 \| TI ("sf 12" or sf12 or "short form 12" or "shortform 12" or "sf twelve" or "short form twelve" or "shortform twelve") or AB ("sf 12" or sf12 or "short form 12" or "shortform 12" or "sf twelve" or "short form twelve" or "shortform twelve") \| Search modes - Find all my search terms \| 475 \| \| S18 \| TI ("sf 36" or sf36 or "short form 36" or "shortform 36" or "sf thirtysix" or "sf thirty six" or "short form thirty six" or "short form thirtysix" or "shortform thirty six" or "shortform thirtysix") or AB("sf 36" or sf36 or "short form 36" or "shortform 36" or "sf thirtysix" or "sf thirty six" or "short form thirty six" or "short form thirtysix" or "shortform thirty six" or "shortform thirtysix") \| Search modes - Find all my search terms \| 2,154 \| \| S19 \| TI ("sf 6d" or sf6d or "short form 6d" or "shortform 6d" or "sf six" or "short form six" or "shortform six") or AB ("sf 6d" or sf6d or "short form 6d" or "shortform 6d" or "sf six" or "short form six" or "shortform six") \| Search modes - Find all my search terms \| 49 \| \| S20 \| hui* \| Search modes - Find all my search terms \| 734 \| \| S21 \| "euro qol" or "euro qol" or eq5d or "eq 5d" \| Search modes - Find all my search terms \| 199 \| \| S22 \| "standard gamble" \| Search modes - Find all my search terms \| 200 \| \| S23 \| "time trade off" or tto \| Search modes - Find all my search terms \| 470 \| \| S24 \| preference* or valuation* \| Search modes - Find all my search terms \| 32,489 \| \| S25 \| S1 OR S2 OR S3 OR S4 OR S5 OR S6 OR S7 OR S8 OR S9 \| Search modes - Find all my search terms \| 20,121 \| \| S26 \| S10 OR S11 OR S12 OR S13 OR S14 OR S15 OR S16 OR S17 OR S18 OR S19 OR S20 OR S21 OR S22 OR S23 OR S24 \| Search modes - Find all my search terms \| 350,056 \| \| S27 \| S25 AND S26 \| Search modes - Find all my search terms \| 1,205 \| \| S28 \| S25 AND S26 \| Limiters - Published Date: 20130101-20181231  Search modes - Find all my search terms \| 499 \| |
| --- | --- | --- | --- | --- | --- | --- | --- | --- | --- | --- | --- | --- | --- | --- | --- | --- | --- | --- | --- | --- | --- | --- | --- | --- | --- | --- | --- | --- | --- | --- | --- | --- | --- | --- | --- | --- | --- | --- | --- | --- | --- | --- | --- | --- | --- | --- | --- | --- | --- | --- | --- | --- | --- | --- | --- | --- | --- | --- | --- | --- | --- | --- | --- | --- | --- | --- | --- | --- | --- | --- | --- | --- | --- | --- | --- | --- | --- | --- | --- | --- | --- | --- | --- | --- | --- | --- | --- | --- | --- | --- | --- | --- | --- | --- | --- | --- | --- | --- | --- | --- | --- | --- | --- | --- | --- | --- | --- | --- | --- | --- | --- | --- | --- | --- | --- | --- |

Database: Ovid PsycINFO 1987 to May Week 4 2018

| 1 chlamydia.mp. or sexually transmitted diseases/ (4408)  2 gonorrhea.mp. or gonorrhea/ (469)  3 ("pelvic inflammatory disease" or PID).mp. (469)  4 cervicitis.mp. (16)  5 ectopic pregnancy.mp. (76)  6 epididymitis.mp. (9)  7 "chronic pelvic pain".mp. (312)  8 Infertility/ or tubal infertility.mp. (1790)  9 tubal factor infertility.mp. (1)  10 life quality.mp. (1514)  11 (hql or qol or HRQL or HRQOL).mp. (13325)  12 (QALY$ or "Quality adjusted Life Year$").mp. (1327)  13 health state$.mp. (1371)  14 (utilit$ or health utilit$).mp. (49734)  15 disutility.mp. (158)  16 (sf 8 or sf8 or "short form 8" or shortform 8 or sf eight or "short form eight" or "shortform eight").tw. (129)  17 (sf 12 or sf12 or "short form 12" or shortform 12 or sf twelve or "short form twelve" or "shortform twelve").tw. (1224)  18 (sf 36 or sf36 or "short form 36" or shortform 36 or sf thirtysix or "sf thirty six" or "short form thirty six" or "short form thirtysix" or "shortform thirty six" or "shortform thirtysix").tw. (4840)  19 (sf 6d or sf6d or "short form 6d" or shortform 6d or sf six or "short form six" or "shortform six").tw. (258)  20 hui$.mp. (1337)  21 (euro qol or euro qol or eq5d or eq 5d).mp. (1826)  22 standard gamble.mp. (202)  23 ("time trade off" or tto).mp. (302)  24 (preference$ or valuation$).mp. (85472)  25 or/1-9 (7064)  26 or/10-24 (152476)  27 25 and 26 [Combined PID Outcomes & HUI concepts from Jackson SR] (220)  28 limit 27 to yr="2013-2018" (76)  29 remove duplicates from 28 (76) |
| --- |
